# Trends and Techniques in 3D Reconstruction and Rendering: A Survey with Emphasis on Gaussian Splatting

**DOI:** 10.3390/s25123626

**Published:** 2025-06-09

**Authors:** Wenhe Chen, Zikai Li, Jingru Guo, Caixia Zheng, Siyi Tian

**Affiliations:** 1 School of Computer Engineering, Jiangsu University of Technology, Changzhou 213001, China; chenwh@jsut.edu.cn (W.C.); 2024655204@smail.jsut.edu.cn (Z.L.); 2 College of Information Sciences and Technology, Northeast Normal University, Changchun 130117, China; guojr228@nenu.edu.cn; 3 School of Electronic Information and Electrical Engineering, Shanghai Jiao Tong University, Shanghai 200240, China

**Keywords:** 3D vision, 3D reconstruction and rendering, 3D Gaussian Splatting, challenges and frontiers

## Abstract

Three-Dimensional Gaussian Splatting (3DGS), an important advancement in the field of computer graphics and 3D vision, has emerged to greatly accelerate the rendering process in novel views’ synthesis. Due to its ability to directly realize the real-time estimation of 3D shapes without neural networks, 3DGS has received a lot of attention in the fields of robotics, urban mapping, autonomous navigation, and virtual reality/augmented reality. In view of the growing popularity of 3DGS, we conduct a systematic review of the relevant literature. We begin by surveying existing work on 3D reconstruction and rendering, outlining the historical development and recent advances from both foundational and innovation-driven perspectives. Next, we summarize the most commonly used datasets and evaluation metrics in 3D reconstruction and rendering. Finally, we summarize the current challenges and suggest potential directions for future research. Through this survey, we aim to provide researchers with a treasure trove of resources in understanding and using techniques related to 3D reconstruction and rendering, in order to promote technological development and application deepening in the field of 3D vision.

## 1. Introduction

**Figure 1:**
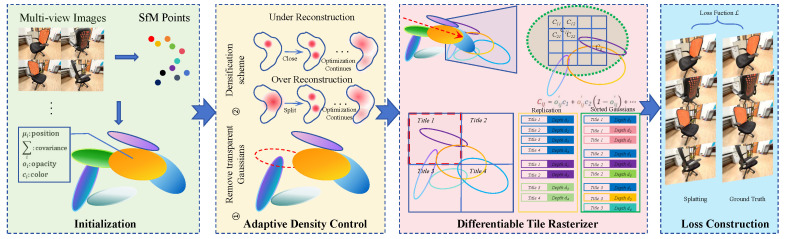
An illustration of the forward process of 3DGS.

In the context of rapid technological development, 3D scene reconstruction and rendering technology has become a key innovation force in the fields of virtual reality (VR), augmented reality (AR), and autonomous driving. Traditional 3D reconstruction methods face many challenges: passive methods are limited by camera accuracy and lighting conditions, resulting in reconstruction distortion; active methods have high accuracy, but expensive equipment and time-consuming data processing consume a lot of computing resources. Deep learning gives rise to reconstruction methods based on point clouds, voxels, meshes, and depth images. However, point cloud-based reconstruction methods have low resolution and incomplete surfaces because of discrete and disorganized point cloud data. Voxel-based methods conflict on high resolution and low computational storage. Mesh-based methods are limited by complex mesh modeling. Depth-image-based methods contain only geometric information and lack color and texture information. As a result, 3DGS technology was developed to provide explicit scene representation, micro-renderable algorithms, real-time rendering capabilities, and editable levels, accurately and quickly representing the geometric scene appearance, compared to the mainstream neural radiation field (NeRF) approach that maps spatial coordinates to pixel values. The 3DGS method takes a sequence of multiview 2D images as input, which are used to initialize a set of 3D Gaussian primitives based on point clouds generated via structure from motion (SfM) [1]. These primitives are then optimized by minimizing the discrepancy between rendered and real images, progressively refining the 3D representation for accurate scene reconstruction. A differentiable chunk-based rasterization technique is introduced to accelerate rendering and improve efficiency. Additionally, an adaptive density control mechanism adjusts the number of Gaussian primitives based on scene complexity, balancing reconstruction quality and computational cost. Figure 1 shows the forward process of 3DGS. Research teams have achieved significant progress with 3DGS in areas such as real-time rendering, image quality enhancement, overcoming large-scale training challenges, improving rendering performance, and supporting autonomous driving. Meanwhile, traditional 3D reconstruction datasets and evaluation metrics have provided support for testing and optimization.

The 3DGS technology is of great significance to the 3D reconstruction field. Firstly, it provides new ideas to solve the limitations of traditional 3D reconstruction methods, reduces the computational complexity and hardware requirements, and enables complex 3D scene processing tasks to run on consumer-grade devices, which provides researchers with more convenient tools and platforms. Secondly, the excellent performance of 3DGS technology in terms of reconstruction effect makes it a comparable choice to cutting-edge methods such as NeRF, which provides researchers with new research directions and technical breakthrough points. In addition, the rapid development of 3DGS technology has spawned a series of innovative results, such as real-time rendering, image quality enhancement, and large-scale training, which not only facilitate the technical iteration of 3DGS, but also expanded its application areas. Researchers can further explore the potential of 3D reconstruction and rendering through these results and promote the innovative development of virtual reality, augmented reality, autonomous driving, and other fields.

Looking ahead, 3DGS technology holds tremendous potential. Future research directions include building large-scale synthetic datasets, exploring self-supervised and weakly supervised learning approaches to reduce dependence on labeled data, and enhancing generalization performance through transfer learning and generative adversarial networks. In addition, combining physical simulation with deep learning may enable high-fidelity reconstruction and rendering of fine-grained details. As an emerging technique, 3DGS is pushing the boundaries of traditional 3D vision and is poised to transform how we perceive and integrate virtual and real-world environments.

**Figure 2:**
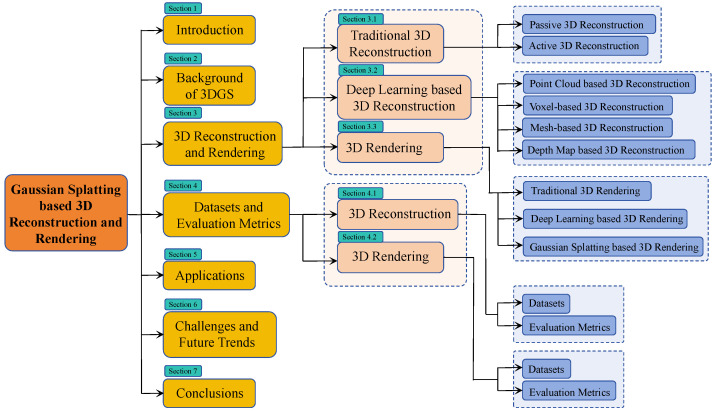
Section Description.

The structure of this investigation is summarized in Figure 2, and the rest of the paper is organized as follows: This paper is a multifaceted discussion around 3D reconstruction and rendering technology. In Section 2, we provide an introduction to the background knowledge on 3DGS. Section 3 presents 3D reconstruction and rendering techniques, beginning with traditional methods, which are categorized into passive and active approaches. Passive methods rely on visual sensors to collect images, whereas active methods emit signals and analyze the returned data to compute 3D information about the target scene. Deep learning-based reconstruction techniques are further categorized by their model representations, including point clouds, voxels, meshes, and depth maps. For rendering, we discuss traditional methods, deep learning-based techniques, and the emerging 3D Gaussian Splatting (3DGS) approach, with a focus on its advantages and improvements over neural radiance fields. In Section 4, we summarize commonly used datasets and evaluation metrics for 3D reconstruction and rendering tasks. Section 5 highlights representative applications of 3DGS across various fields, including cultural heritage, architecture, virtual reality, medicine, and autonomous driving. Section 6 outlines the key challenges facing 3DGS and discusses potential future research directions. Finally, Section 7 provides a comprehensive overview of 3DGS, emphasizing its transformative potential in explicit radiance field modeling, computer graphics, and computer vision, and its expected future role in advancing 3D reconstruction technologies. While we emphasize its transformative potential in graphics and vision, we also acknowledge its current constraints, such as its high memory consumption, limited scalability to large dynamic scenes, and challenges in real-time optimization, providing a more balanced perspective.

## 2. Background of 3DGS

**Traditional point-based rendering.** Point-based rendering technique renders a set of clear shape units to produce more realistic images. On the basis of point-based rendering, Zwicker et al. [2] proposed an elliptical splat-rendering technique for occupying multiple pixel points, which enhanced the hole-free image generation by mutual coverage of neighboring splats. Kopanas et al. [3] innovatively proposed a point-based differentiable channel that was capable of fusing bi-directional elliptical weighted splats. In recent research, a large number of cutting-edge advances such as texture filters for antialiased rendering [4], improving rendering efficiency [5], and solving discrete shading [6] have been successively proposed.

Traditional point-based rendering methods focus on producing high-quality rendering for a specific geometry. Solving point-based rendering using a neural implicit representation is gaining popularity among a large number of researchers. The method does not rely on a predefined geometry for 3D reconstruction purposes. A representative work is neural radiation field (NeRF) [7], which utilizes an implicit density field to model the geometry and employs a shape field to estimate the view-dependent color $c_{i}$. Point-based rendering fuses all the points sampled along the camera rays to generate the pixel color *C*, as shown in Equation (1).

(1) $$C=\sum_{i=1}^{N}c_{i}\alpha_{i}T_{i}$$

where *N* denotes the number of sampling points. The color and opacity value of the *i*th point on the light is denoted as $\alpha_{i}=\exp\left(-\sum_{j=1}^{i-1}\sigma_{j}\delta_{j}\right)$. Here, $\sigma_{j}$ is the density value of the *j*th point. The cumulative transmittance is given by $T_{i}=\prod_{j=1}^{i-1}\left(1-\alpha_{j}\right)$. NeRF converts the density values to opacity values, while the rendering process of 3DGS directly models the opacity values. Notably, 3DGS uses raster-based rendering without sampling.

**Gaussian Spallation Radiation Field.** The Gaussian scattering radiation field is an explicit scene representation of a radiation field [8]. It uses the radiation field of a large number of 3D anisotropic spheres, each of which uses a 3D Gaussian distribution (Equation (2)). Specifically, each anisotropic ball has a mean $M\in R^{3}$, covariance ∑, opacity α∈R, and a ball-harmonic parameter $C\in R^{k}$, where *k* is the degree of freedom. To regularize the representation, Equation (3) decomposes the covariance matrix into a rotation matrix *R* and a scaling matrix *S*. They are further represented as quaternions $r\in R^{4}$ and scaling factors $s\in R^{3}$.

(2) $$G\left(X\right)=e^{-\frac{1}{2}M^{T}\Sigma^{-1}M}$$

(3) $$\Sigma =RSS^{T}R^{T}$$

View rendering is executed by point-splash [9] enforcement. Specifically, all Gaussian spheres in the scene are projected onto the 2D image plane and their colors are computed using the sphere harmonics. For each 16×16 pixel block of the image, the Gaussian projections intersecting the block are ordered by depth value. For every pixel in the patch, its color is computed by alpha compositing the opacity and color of all the Gaussians covering this pixel by depth order, as shown in Equation (4).

(4) $$C=\sum_{i\in N_{\mathrm{cov}}\;}c_{i}\alpha_{i}\prod_{j=1}^{i-1}\left(1-\alpha_{j}\right)$$

where $N_{\mathrm{cov}}$ denotes the splats covering the pixel, $alpha_{i}$ denotes the opacity of the Gaussian Splatting multiplied by the density of the 2D Gaussian distribution projected at the pixel location, and $c_{i}$ denotes the color.

## 3. Three-Dimensional Reconstruction and Rendering

In the field of autopilot, 3D reconstruction and rendering are key technologies focusing on the comprehensive reconstruction of the whole scene. Three-dimensional reconstruction aims at recovering the 3D information of an object or scene from various types of input data (e.g., 2D images, point clouds, etc.) and thus constructing its digital model. Three-dimensional rendering is the process of transforming 3D models and scenes into 2D images or videos. Therefore, 3D reconstruction and rendering are not only a simple combination of 3D models of individual objects but also consider the spatial relationship between multiple objects in the scene, lighting conditions, occlusion, and other complex factors to create a complete and realistic virtual scene. Especially in cutting-edge fields such as virtual reality, augmented reality, and autonomous driving, 3D reconstruction and rendering play an indispensable role. The following section introduces the research lineage and recent progress of 3D scene reconstruction and rendering in detail.

### 3.1. Traditional 3D Reconstruction

Classified according to the way of acquiring input data, 3D scene reconstruction can be divided into passive 3D reconstruction and active 3D reconstruction. Passive 3D reconstruction acquires images through camera equipment and then analyzes and processes the images using the principle of multiview geometry to obtain the 3D information of the object or scene. Active 3D reconstruction realizes the 3D model reconstruction of the object by actively detecting the depth information of the environment, such as LiDAR and structured light scanning. The following section provides a brief introduction to the related technologies.

#### 3.1.1. Passive 3D Reconstruction

Passive 3D reconstruction captures images from visual sensors (e.g., cameras) and uses stereo matching or other geometric analysis methods to recover the 3D structure of an object or scene from multiple viewpoints. The method is inexpensive, simple, and suitable for complex scenes. Classified according to the number of cameras, passive 3D reconstruction can be categorized into monocular vision, binocular vision, and multicamera 3D reconstruction methods.

Monocular vision utilizes a single camera device to collect images and reconstructs the 3D model by extracting texture, contour, and other features of the image, which has the advantages of being low cost and a simple operation. In the following section, the classical light-and-dark shape-restoring method, photometric stereo vision method, and motion-restoring structure method are described.

In 1970, Horn [10] proposed the Shape-from-Shading (SFS) method, which utilized the light and dark variations on an object surface in an image to recover the object 3D contour. Fan et al. [11] developed an SFS model based on the Cook–Torrance bi-directional reflectance distribution function and experimentally demonstrated its accuracy and robustness. In 2010, Woodham [12] improved the light-and-dark shape-recovery method and proposed the Photometric Stereo (PS) method. That method solved the 3D information of the surface of the observed object by changing the relationship between the incident light direction and the light and dark of the image. Later, Shi et al. [13] proposed a self-calibrating photometric stereo vision method that solved the double-calibration problem in standard photometric stereo algorithms by analyzing the color or intensity distributions in the image and irradiance domains. Morris et al. [14] introduced a novel stereo matching criterion applicable to refractive scenes as well as an algorithm based on a dynamically refractive stereo method that produced detailed 3D reconstruction results that captured dynamic behavior. To deal with the problem of non-Lambertian reflections, Shen et al. [15] proposed a kernel regression-based photometric stereometry method that improved computational efficiency by determining the optimal parameters of the variable kernel through fast cross-validation, matrix computation, and proper normal initialization. In addition, the shape-from-texture (SFT) method, the shape-from-silhouettes/contours (SFS/SFC) method, or the “silhouette method” for short, and the shape-from-focus (SFF) method are also available. These methods rely on analyzing the surface details (e.g., reflected light, texture, contour shape, etc.) in the image to recover the shape changes or details of the object surface. However, in practice, these methods do not have high reconstruction accuracy in some scenes or on specific objects or cannot be reconstructed.

**Figure 3:**
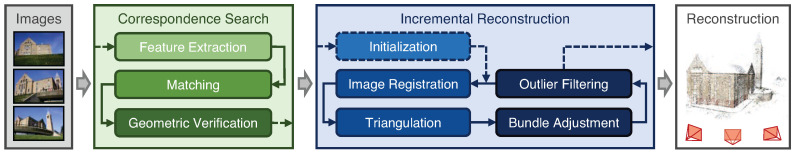
SFM process structure [1].

Structure from motion (SFM) is a technique to recover the camera position and 3D scene structure from multiple images. SFM obtains the correspondence of the same pixel points in the image through a matching algorithm and utilizes the matching constraint relationship and the principle of triangulation to obtain the 3D coordinates of the target object and realize the 3D scene reconstruction, which is shown in Figure 3. At present, SFM is mainly categorized into global SFM, incremental SFM, and hybrid SFM.

Global SFM involves adding all images to the scene to estimate the camera pose and then triangulating them. This method can obtain the camera position and scene structure at once. Global SFM is characterized by its fast reconstruction speed and high accuracy but poor robustness. Global SFM was first proposed by Sturm et al. [16] in 1996. In order to generate a better and robust model, Crandall et al. [17] proposed a Markov Random Field-based SFM formulation which combined the camera and point sources of information. Moulon et al. [18] proposed a global calibration method based on the relative motion between image pairs as a way to extract precise translation directions and accurate camera positions. Inspired by nested anatomical algorithms, Zhu et al. [19] proposed a distributed and robust motion-averaging method that handled the large-scale motion problem in a distributed manner and re-optimized the camera positions so that global SFM could be used at the scale of millions of images.

The incremental SFM method progressively triangulates and computes the camera pose by matching feature points on the image, while optimizing it using local beam method leveling (Bundle Adjustment, BA). Although the method has high robustness, its computational efficiency is low. In addition, due to the presence of cumulative errors, incremental SFM is prone to drift problems, especially in the absence of global optimization. For this reason, Wu [20] achieved a balance between speed and accuracy for incremental SFM and reduced the feature matching cost of large-scale SFM by introducing a new BA strategy. To solve the problems of existing SFM in 3D reconstruction, Schonberger et al. [1] proposed a new optimization method for each step in incremental SFM reconstruction, which improved the completeness, robustness, and accuracy of the scene reconstruction. Wang et al. [21] proposed a two-view structure-from-motion (SfM) framework that integrated deep learning with traditional geometric methods, leveraging optical flow estimation, normalized pose estimation, and scale-invariant depth estimation to improve camera pose and depth estimation accuracy, outperforming existing methods on multiple datasets. Qu et al. [22] proposed an outdoor reconstruction algorithm for UAV images by selecting key UAV images to create an image queue and then using incremental SFM to compute the queue images, which enabled incremental SFM to be used for large-scale outdoor scene reconstruction. After that, Cui et al. [23] proposed a large-scale outdoor scene reconstruction method based on linear incremental SFM, which effectively improved the efficiency and quality of 3D reconstruction of blurred images.

Hybrid SFM [24] is an effective fusion of the advantages of global SFM and incremental SFM, which uses global SFM to estimate the camera rotation and incremental SFM to compute the estimated camera center coordinates, thus solving the problems of efficiency, robustness, and accuracy. Zhu et al. [25] used the relative motions in the incremental SFM to globally average the camera bit position, which improved the accuracy of the algorithm.

Binocular vision technology reconstructs the 3D model of an object by simulating the parallax principle of the human visual system and utilizing the parallax relationship between the two camera devices for depth calculation. The processing flow of binocular vision mainly includes five steps: image acquisition, camera calibration, image correction, stereo matching, and 3D reconstruction.

In stereo matching, depth information is obtained by finding matching pixel points in two images and calculating the parallax value. Scharstein et al. [26] evaluate the stereo matching algorithms in detail and proposed four core components for dense stereo matching: matching costing, cost aggregation, parallax calculation and optimization, and parallax improvement. By systematically categorizing these steps, the direction of algorithm optimization for practical applications was guided. Huang et al. [27] proposed a correction, matching, and 3D reconstruction method for binocular stereo vision systems, which combined a reconstruction error-based correction and a semiglobal matching algorithm with least-squares fitting and effectively improved the accuracy of the parallax maps, which in turn enhanced the robot’s autonomy and intelligent level. Wei et al. [28] applied the 3D reconstruction technique of binocular vision to a remote-controlled excavator and solved the problem that the remote-controlled excavator could not perceive the stereo distance of the target in video surveillance by removing the noise generated by binocular cameras in data acquisition, optimizing the matching of image features, and improving the parallax map processing. Multicamera vision uses three or more cameras to photograph the object from different viewpoints, which expands the field of view and reduces the blind zone through multiple viewpoints, improving the matching accuracy and reconstruction precision. For example, Xu et al. [29] proposed a real-time 3D measurement system for the harsh environment (high temperature, high dust, and no light) in the blast-furnace ironmaking process. The system utilized industrial endoscopes to acquire material surface images, and realized real-time, high-precision reconstruction of the 3D shape of the blast furnace’s material surface by acquiring its 3D information in order to construct a virtual binocular camera array.

**Figure 4:**
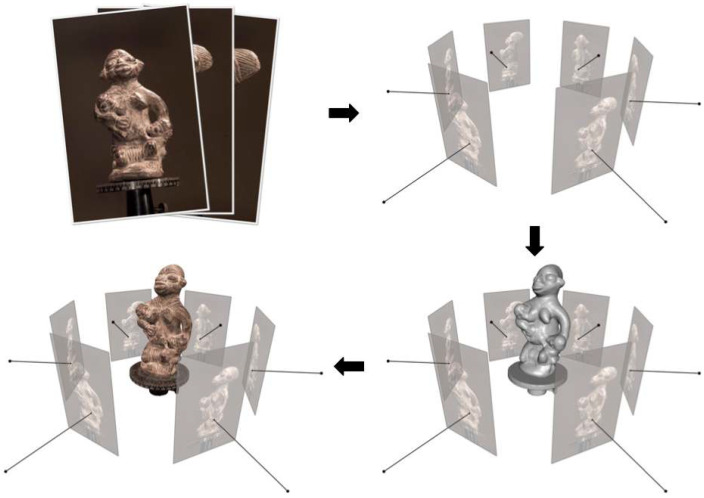
Multiview stereo example. Clockwise representation: input image, placed image, reconstructed 3D geometry, textured geometry [30].

Multi-vision technology is dedicated to mining the 3D information of the scene from the images of multiple viewpoints, among which the multiview stereo (MVS) technology is especially attractive. Different from the SFM method, MVS technology captures images of the same scene from different angles simultaneously by multiple cameras, processes and analyzes the images using computer vision algorithms to calculate the 3D coordinates of the objects, and finally realizes the 3D reconstruction of the scene. Compared with binocular vision, MVS synthesizes the parallax information between images from multiple different viewpoints, which can achieve higher accuracy and more complete 3D reconstruction, and an example of multiview stereo is given in Figure 4 (figure obtained from [30]). Furukawa and Ponce [31] proposed a face-slice-based multiview stereo vision (patch-based MVS) method, which automatically generated dense 3D models from multiple images and solved the problem of 3D reconstruction under no initialization conditions. Shen [32] proposed a large-scale scene MVS method based on depth map merging, which generated depth maps with acceptable errors by using an efficient block-based stereo matching process and then obtained a more accurate and dense point cloud by depth map refinement techniques. Zheng et al. [33] proposed a multiview depth map estimation method based on PatchMatch, which was able to adaptively determine the pixel-level data associations between the reference image and the source image set, overcoming the data association problem in multiview depth map estimation, and thus improving the accuracy of depth estimation.

Recently, novel methods have emerged that leverage single panoramic images for immersive 3D reconstruction. For example, Jashari et al. [34] evaluated AI-based static stereoscopic rendering techniques for indoor spherical imagery, while Pintore et al. [35] proposed PanoVerse, which automatically generated stereoscopic 3D environments from a single panorama. These approaches represent a significant shift in passive 3D reconstruction, enabling scene understanding and immersive rendering with minimal input data.

#### 3.1.2. Active 3D Reconstruction

Active 3D reconstruction methods utilize sensors to actively emit a signal source to the surface of an object and calculate the 3D information of the target object by parsing the returned signal. According to the method principle, active 3D reconstruction can be broadly categorized into structured-light-based ranging and laser-based ranging, and the following briefly introduces several commonly used methods. In 3D reconstruction, the structured-light-based method utilizes the principle of triangulation to obtain depth information by calculating the triangulation between the projection device, the image acquisition device, and the object under test. For example, Gunatilake et al. [36] proposed a solution for real-time scanning and reconstruction of 3D contours of pipelines using a tracked robot, which generated high-precision 3D maps in real time by using algorithms such as stereo mapping, light projection, and RGB depth mapping. Montusiewicz et al. [37] used a 3D scanner with structured-light technology to digitize historical costumes by creating 3D models of historical costumes to promote the documentation and dissemination of costume culture. Chen et al. [38] proposed a reinforcement learning (RL)-based next-best-view (NBV) policy that overcame the limitations of hand-crafted criteria and per-scene optimizations by introducing a 5D free-space action framework and multi-source state embeddings, achieving superior generalizability across datasets in active 3D reconstruction.

The Time-of-Light (TOF) method calculates the distance to the target object by transmitting light pulse signals to the object and calculating the distance to the target object based on the time difference between the reflections of the light pulse signals received by the sensor. This method has the advantage of working in light-free environments and is widely used for 3D reconstruction due to its high measurement stability. Guðmundsson et al. [39] used a TOF camera in a smart room to achieve the 3D reconstruction of foreground targets by combining the background probabilistic model to segment the foreground from the background in the view. To improve the efficiency and safety of car wash systems, Hoegg et al. [40] proposed a concept of automatic, non-contact online vehicle 3D measurement, which enabled fast and efficient 3D point cloud reconstruction by merging and synchronizing data acquired by multiple TOF cameras. Li et al. [41] proposed a delay-based correction method that was simple to operate and fast to calibrate, thus significantly improving the TOF camera’s linearity and accuracy.

LiDAR acquires high-precision 3D point cloud data by transmitting a laser beam and measuring the time difference from laser emission to reception. These point cloud data can realize the 3D reconstruction of the target object after preprocessing, feature extraction, and other steps. Currently, LiDAR is widely used in terrain mapping [42], autonomous driving [43], etc. Schwalbe et al. [44] proposed a 3D reconstruction method that combined point cloud data with Geographic Information System (GIS) data. The method utilized LiDAR to acquire the point cloud data of a building, then rotated the point cloud into a specific orthogonal projection for processing, and identified the roof structure in the projection to reconstruct a 3D model of the building. To meet the challenges of a large number of point clouds, a complex data structure, and multiclass targets, Feng et al. [45] proposed a point cloud processing method utilizing dual-channel airborne LiDAR data, which not only improved the performance of extracting building instances but also reduced the computational burden. Zhong et al. [46] proposed a LiDAR scanning technique for dynamic environments, which continuously acquired dynamic point cloud data and segmented the dynamic objects to achieve the accurate reconstruction of the outdoor environment. A recent study by Tukur et al. [47] provides a comprehensive comparison between passive and active reconstruction pipelines within virtual staging environments. The work highlights key trade-offs in cost, fidelity, and real-time capability, further validating the relevance of hybrid or task-specific strategies in 3D scene modeling.

### 3.2. Deep Learning-Based 3D Reconstruction

Traditional 3D reconstruction pipelines, such as structure-from-motion (SfM) and multiview stereo (MVS), rely on hand-crafted features, multi-stage optimization, and are often sensitive to variations in viewpoint, illumination, and image quality. COLMAP, as a representative implementation, integrates robust SfM and dense MVS techniques [1]. Classic evaluations of MVS algorithms have highlighted their dependence on high-quality, densely sampled images and their performance bottlenecks in low-texture or occluded regions [48].

With the advancement of deep learning, end-to-end MVS frameworks such as MVSNet [49] have emerged, demonstrating improved robustness by leveraging semantic features learned from data. Neural rendering methods like neural radiance field (NeRF) [7] further extend this trend by implicitly modeling geometry and appearance, enabling high-quality view synthesis from sparse inputs. These learning-based methods have shown promising performance in addressing certain limitations of traditional reconstruction pipelines, particularly in handling textureless regions, occlusions, and complex lighting. However, they also face challenges such as data dependency, limited generalization across diverse scenes, and high computational demands. A comprehensive tutorial by Furukawa and Hernández [30] provides an in-depth analysis of the evolution, challenges, and limitations of MVS methods, forming the foundation for many modern learning-based approaches.

**Figure 5:**
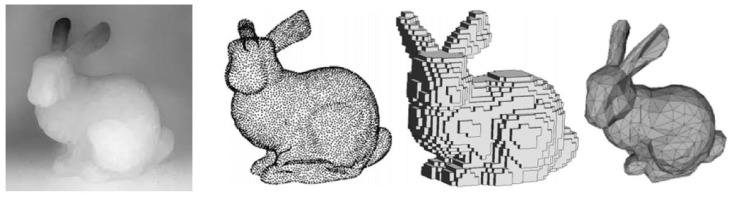
The four representations of a 3D model. From left to right: depth map, point cloud, voxel, and mesh.

According to the representation of 3D scene reconstruction model, the 3D reconstruction based on deep learning can be categorized into 3D reconstruction based on point cloud, 3D reconstruction based on voxels, 3D reconstruction based on a mesh, and 3D reconstruction based on depth map, and Figure 5 demonstrates four of the representations of the 3D model. In the following section, we provide a detailed introduction to each of the four deep learning-based 3D reconstruction models mentioned above.

#### 3.2.1. Point Cloud-Based 3D Reconstruction

A point cloud is a collection of discrete points on the surface of an object using 3D coordinates, colors and other information. Point cloud-based 3D reconstruction methods consume less memory and the shape of the reconstructed model is smooth. However, when 3D reconstruction is performed on a single image, it usually faces the problem of blur and incompleteness. To address this challenge, Chen et al. [50] proposed Point-MVSNet, a network framework that directly processed the target scene into a point cloud, which fused 3D geometric a priori and 2D texture information for improving the reconstruction accuracy of the point cloud with faster computational efficiency and greater flexibility. Huang et al. [51] proposed an unsupervised multimetric multiview stereo vision network, M3VSNet, which learned intrinsic constraints from different matching perspectives, improved the completeness of point cloud reconstruction, and solved the problems of traditional supervised learning relying on depth maps as well as the poor robustness of unsupervised methods. Nichol et al. [52] explored an alternative approach to 3D target generation, which first generated a single synthetic view using a text-to-image diffusion model and then generated the view into a 3D object using a second diffusion model to generate the view into a 3D point cloud. Li et al. [53] proposed a Transformer-based point cloud completion network, ProxyFormer, which took incomplete point cloud data as inputs and utilized a proxy point cloud to assist in completing the point cloud completion. Zhang et al. [54] proposed an RA-MVSNet approach that utilized point-to-surface distances to the surface so that the model could perceive a wider range of surfaces, resulting in a more complete reconstruction. Koch [55] proposed a method for 3D scene graph prediction with a self-supervised pre-training method, SGRec3D, which exploited large-scale 3D scene understanding datasets by reconstructing the 3D input scene from graph bottlenecks as a pre-training task so that pre-training did not require object-relational labeling.

Point cloud-based 3D reconstruction methods represent object shapes in terms of unordered point sets, avoiding complex structure maintenance, and can effectively capture the local and global features of objects when dealing with large-scale point cloud data, with high adaptability to irregular shapes. However, the discrete nature of the point cloud makes the reconstructed 3D model surface incomplete and low resolution.

#### 3.2.2. Voxel-Based 3D Reconstruction

**Figure 6:**
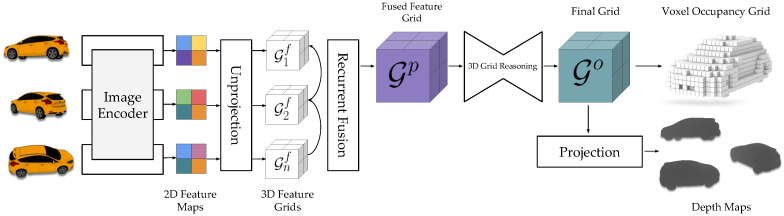
Flowchart of voxel-based multiview stereo vision network [57].

Voxel is similar to the smallest unit pixel in 2D space and is the smallest unit on the 3D spatial segmentation. A common approach is generally to use an encoder–decoder approach for 3D reconstruction. Ji et al. [56] proposed an end-to-end MVS vision learning framework, SurfaceNet, which took a set of images and their camera parameters as inputs to directly learn photographic consistency and geometric relationships of surface structures to achieve end-to-end multiview stereo vision, thus predicting the 3D model. Kar et al. [57] proposed a learning system, LSM, for MVS, which utilized feature projection and backprojection along the line of sight to reflect pixel features to the voxel mesh to determine whether a voxel belonged to the object surface or not, to achieve end-to-end learning to accomplish the metric 3D reconstruction task; Figure 6 shows the flowchart of the voxel-based multiview stereo vision network. Liu et al. [58] proposed a generative model of a variational shape learner, which learned the voxelized 3D model in an unsupervised manner to learn the latent structure of voxelized 3D shapes and used an inverse convolutional network to decode the learned global and local latent codes into voxels to reconstruct the voxel model. Wang et al. [59] formulated the multiview 3D reconstruction as a sequence-to-sequence prediction problem and proposed a multiview 3D reconstruction framework based on a Transformer, putting feature extraction and view fusion in the Transformer, which improved the accuracy and performance of voxel-based 3D reconstruction methods. Schwarz et al. [60] proposed a VoxGRAF model, which took the sparse voxel generator as the core and generated the radiation field on the sparse voxels to achieve efficient and high-quality scene reconstruction and image synthesis, as shown in Figure 6.

Voxel-based 3D reconstruction methods divide the 3D space into regular voxel grids, a representation that facilitates parallel computation and efficiently handles voxel-level operations. However, the voxel representation usually has limited resolution, and high-resolution voxel modeling leads to huge storage and computation overheads.

#### 3.2.3. Mesh-Based 3D Reconstruction

**Figure 7:**
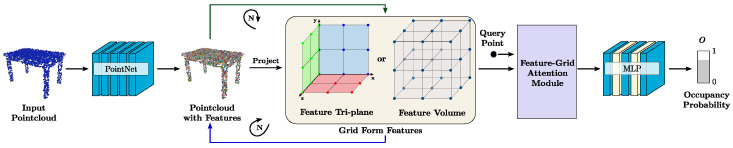
Three-dimensionalmesh model based on alternating potential topology [64].

A mesh is a structure formed by meshing data such as images or 3D models, which consists of neighboring point clouds forming polygonal mesh data; compared to voxel and point cloud models, mesh models can completely represent the surface shape of the target object. Wang et al. [61] proposed a pyramid-structured mesh-guided MVS method, which first utilized the PatchMatch algorithm to generate a depth map for coarse-scale images and obtain a surface mesh, then projected the mesh to the depth map to replace unreliable depth values, and input the corrected depth map into the fine-scale reconstruction for initialization to obtain a complete 3D model. Yuan et al. [62] proposed a view-attention-guided network, VANet, which introduced a channel view-attention mechanism and a dual-pathway network architecture, which improved the quality of single-view and multiview 3D reconstruction under a unified framework to generate high-quality 3D meshes. Ju et al. [63] proposed a real-time 3D scene reconstruction method, DG-Recon, which solved the feature fusion problem in monocular image reconstruction through deep a priori guidance and improved the problems of low reconstruction accuracy, incomplete geometry, or high computational cost, and the inability of existing methods to be processed in real time. Wang et al. [64] proposed an Alternating Latent Topologies (ALTO) method, which fused the advantages of different latent topologies through a latent alternation strategy and attention-based coding to realize high-fidelity implicit 3D indication reconstruction and transform it into a 3D mesh model, as shown in Figure 7. For the limitations existing in single-view 3D reconstruction, Dogaru et al. [65] proposed a hybrid approach with a divide-and-conquer strategy, which extracted depth and semantic information, then reconstructed the components with a single object-level method, and achieved complete reconstruction of a single view by combining the processing, which demonstrated better reconstruction performance than previous work in synthetic and realistic scenarios.

Mesh-based 3D reconstruction methods utilize triangular or polygonal meshes to represent the surface of an object, which can accurately describe the shape and topology of the object, generate high-quality, smooth surface models, conform to human’s intuitive understanding of the shape of the object, and are widely used in the field of graphics rendering and animation production. However, the mesh structure is complex and requires well-designed algorithms to deal with topological changes and deformations, which makes the modeling process relatively complex and computationally expensive.

#### 3.2.4. Depth Map-Based 3D Reconstruction

**Figure 8:**
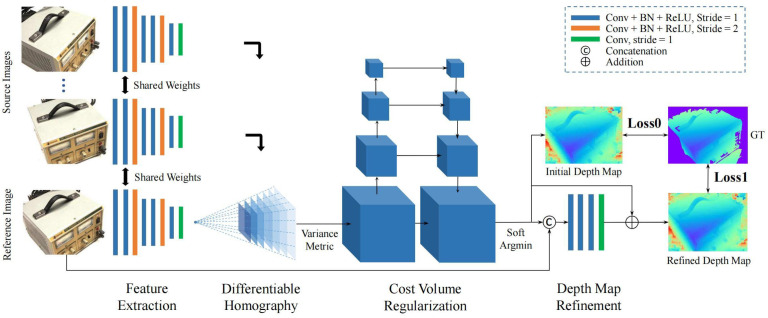
MVSNet network framework [49].

Depth Map-based 3D scene reconstruction methods essentially recover the 3D scene of an object or scene step by step by using the depth value of each pixel in the depth map, combined with the parameters and coordinate relations of the camera. Depth map-based methods transform the 3D reconstruction problem into a depth estimation problem and can transform the depth map into point clouds, meshes, voxels, etc. Huang et al. [66] proposed DeepMVS, a deep convolutional neural network for multiview stereo reconstruction, which solved the deficiencies of traditional MVS algorithms in dealing with untextured regions and thin structures by integrating multiview information. Yao et al. [49] proposed an end-to-end MVSNet method by combining convolutional neural networks with MVS. This method extracts the depth features of an image through the network, determines the accumulated cost using a differentiable monoreactivity matrix, and then performs regularization using 3D convolution for depth estimation; Figure 8 shows the network framework of MVSNet. Yi et al. [67] proposed the pyramidal MVS network, PVA-MVSNet, which constructed an image pyramid with multiple scales and used adaptive view aggregation by obtaining depth maps at different scales and then corrected the mismatch error with reliable depth propagation from low-resolution images to high-resolution images to obtain an accurate depth map, thus solving the problem of dealing with matching ambiguity in MVS. Zhang et al. [68] proposed a geometric perception model, GeoMVSNet, which used geometric-prior-guided feature fusion, probabilistic volumetric geometric embedding, frequency-domain geometric enhancement, and depth-distributed similarity loss, achieved more accurate depth estimation and 3D scene reconstruction, and solved the problem that existing MVS methods did not fully utilize the geometric information in depth estimation leading to fragile cost matching and poor reconstruction results.

Although depth map-based 3D reconstruction methods utilize the complementarity between depth maps from different viewpoints to improve the quality of reconstruction, the depth map only contains geometric information of the object and lacks color and texture information, which makes the final 3D model visually monotonous and affects the visual effect.

### 3.3. Three-Dimensional Rendering

Three-dimensional rendering is the process of converting a 3D model obtained by 3D reconstruction methods into a 2D image, and the specific rendering method is determined by the representation form of the 3D model. The representation of 3D reconstruction model can be divided into explicit representation and implicit representation. Explicit representation mainly records the 3D model in data formats such as meshes, point clouds, voxels, and parametric surface functions, etc. Implicit representation stores the 3D model in the data format mainly based on neural radiation fields and implicit surface functions. Currently, 3D models based on explicit representation are mainly rendered using traditional graphics theory. While neural network rendering is mainly oriented to implicitly represented 3D models, the rendering process of graphics is simulated through neural network learning to realize end-to-end mapping. The following section introduces the commonly used rendering methods from traditional methods, deep learning methods, and 3D Gaussian Splatting (3DGS).

#### 3.3.1. Traditional 3D Rendering

Rendering methods based on traditional methods achieve the conversion of a 3D model from a 3D space to a 2D plane through mathematical modeling, geometric calculations, and the simulation of physical optics principles. The following section describes common ray-tracing and rasterization rendering methods. Ray tracing simulates the propagation and reflection of light to reverse the path of light and reconstruct the 3D structure of an object or scene. This method is computationally efficient and plays an important role in the fields of gaming, virtual reality (VR), digital twins, etc. Dib et al. [69] proposed a face reconstruction method based on microscopic ray tracing, which modeled the illumination of the scene by parameterizing the virtual light steps and introduced an optimization method from coarse to fine to achieve face reconstruction. Zhang et al. [70] proposed a Quantum Correlated Ray-Tracing Imaging (QRTI) technique to reconstruct the propagation trajectory of photons by capturing two photons in different image planes using a time-tagged camera and utilizing the position, momentum, and time dependence of the photons. It resulted in a more accurate and realistic rendering of the 3D scene.

Rasterization rendering is a method of projecting objects in a 3D scene onto a 2D screen. The rasterization process is divided into two main steps: the first step requires the projection of objects in space onto the imaging plane, and the second part detects whether the objects in the projected region are in the region and fills them with the corresponding color if they exist. Yifan et al. [8] proposed a point-based Differentiable Surface Splatting (DSS), which improved on the traditional rasterization rendering method and employed elliptic weighted average filtering to process the point attributes in order to optimize the point cloud rendering effect and achieve high-quality 3D reconstruction and rendering. To address the efficiency and quality of point cloud rendering, Ma et al. [71] use rasterization to achieve effective point cloud searching and sampling to improve the speed and quality of point cloud rendering. In addition, that method is often combined with the ray-tracing method to solve the problem of lack of detail, thus realizing high-fidelity rendering.

#### 3.3.2. Deep Learning-Based 3D Rendering

Deep learning-based rendering methods obtain implicit 3D information from neural networks and synthesize it using graphical rendering principles to finally output the rendered results. Anciukevičius et al. [72] first proposed the diffusion model RenderDiffusion for 3D generation and inference, which converted a monocular two-dimensional image into a three-dimensional representation of the scene, used a multilayer perceptron (MLP) to convert the three-plane features into density and color, and finally used the body rendering integral to get the pixel color for rendering. Zhou et al. [73] proposed SparseFusion, a sparse view 3D reconstruction method which combined the Epipolar Feature Transformer and View-conditioned Latent Diffusion Model to model the new view image distribution and reconstructed the corresponding 3D implicit field using diffusion distillation to render an image with arbitrary viewpoints. An et al. [74] proposed the 3D perceptual generation model PanoHead, which could reconstruct a 360-degree full-head high-quality, view-consistent 3D model, solving the limitations of 3D GANs to synthesize 3D heads. PanoHead uses foreground perception to discriminate the foreground from the background and generates a detail-rich 3D model by tri-grid feature sampling and by combining the camera position to achieve multi-angle rendering.

**Figure 9:**
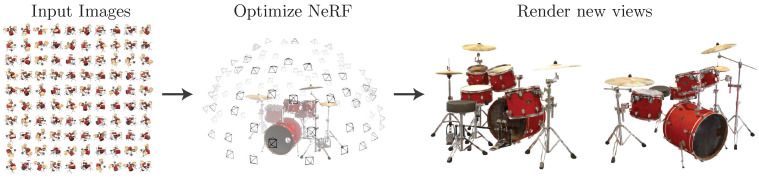
The overall flow of the NeRF methodology [7].

A neural radiance field (NeRF) [7] is a neural network scene representation for implicit space, which reconstructs a detailed and realistic 3D scene by predicting information such as color and density using a neural network. The NeRF method has become a popular new view synthesis method, which observes the scene at a known viewpoint to synthesize arbitrary images in new viewpoints, and the overall process is shown in Figure 9. Mirzaei et al. [75] proposed the SPIn-NeRF method, which utilized a NeRF to obtain a 3D-consistent mask under single-view sparse annotations to accurately reconstruct the 3D scene. The view-consistent high-quality 3D model rendering was then realized using the NeRF-based ray sampling and body rendering mechanism as well as combining the RGB prior and depth prior. To address the challenge of training NeRFs with eye pre-computed camera poses, Bian et al. [76] proposed the NoPe-NeRF method, which reconstructed an accurate 3D scene by jointly optimizing the camera poses and the NeRF method. In this, the NeRF-based rendering mechanism converted the 3D scene into a 2D image and combined the corrected depth information to achieve a high-quality and view-consistent new view image. Wynn et al. [77] proposed the DiffusioNeRF method, which solved the problem of NeRF scene geometry and color fields. The method optimized the density and color fields based on NeRF, while learning the scene geometry and color prior using the Denoising Diffusion Model (DDM), which improved the quality of new view synthesis and the accuracy of 3D reconstruction.

#### 3.3.3. Gaussian Splatting-Based 3D Rendering

The 3D Gaussian Splatting-based rendering method (3DGS) is a Gaussian function-based scene reconstruction and real-time 3D rendering technique, which realizes efficient 3D reconstruction and rendering by transforming point cloud data or particles in the scene into the form of Gaussian functions. Compared to the traditional NeRF (neural radiation field) method, 3DGS has the advantages of fewer computations, low memory consumption, and high-quality reconstruction results, which make it show a wide range of application potential in the fields of computer graphics, computer vision, and virtual reality.

By using Gaussian functions to represent points in the scene, 3DGS is able to efficiently deal with complex geometric structures and illumination changes. Compared with NeRF, 3DGS has obvious advantages in real-time rendering and editability. For example, Wu et al. [78] introduced 4D Gaussian Splatting (4D-GS), a novel representation combining 3D Gaussian functions and 4D neural voxels to efficiently render dynamic scenes in real time, achieving 82 FPS at 800×800 resolution while maintaining high visual quality. In addition, 3DGS performs well in processing high-resolution images and is able to generate detailed 3D models [79]. In terms of target representation, 3DGS is able to accurately capture the shape and texture information [80] of targets by optimizing the Gaussian distribution parameters. Zhang et al. [81] introduced a scalable transformer-based model that predicted high-quality 3D Gaussian primitives from sparse posed images, enabling fast and accurate 3D reconstruction for both objects and scenes while outperforming state-of-the-art methods. For example, in medical image processing, 3DGS can be used to accurately reconstruct organs and diseased areas to assist doctors in diagnosis and surgery planning [82].

Gao et al. [83] proposed a 3D point cloud rendering method for decomposing materials and lighting from multiview images, which combined with ray tracing techniques for boundary volume hierarchies to achieve realistic lighting effects and efficient real-time rendering. Lee et al. [84] manipulated the covariance of 3D Gaussian functions through a small multilayer perceptron to achieve real-time deblurred rendering, which improved the image quality while maintaining a fast rendering speed. Liu et al. [85] proposed the CityGaussian method, which employed a divide-and-conquer training strategy and a detailed hierarchical strategy to achieve efficient training and high-quality rendering of large-scale scenes and was capable of rendering large-scale scenes at different scales in real time. Yu et al. [86] solved the artifact problem of 3DGS when the sampling rate varies by introducing a 3D smoothing filter and a 2D Mip filter. The 3D smoothing filter avoided artifacts caused by high-frequency information by limiting the frequency of 3D Gaussian primitives; the 2D Mip filter replaced the 2D expansion filter in 3DGS to avoid light diffusion and improved the realism of rendering, which improved the rendering effect and generalization ability of 3DGS in different scenes.

To improve the tracking accuracy and rendering quality of SLAM (simultaneous localization and map construction), Sun et al. [87] proposed the MM3DGS SLAM method. The method built a multimodal SLAM framework based on 3DGS and realized real-time rendering and accurate trajectory tracking by fusing visual, inertial, and depth information. Among them, 3DGS achieved image rendering through feature computation and rasterization, thus generating images with rich details and fewer artifacts. With the in-depth research on 3DGS, methods such as those in [88,89,90,91] have attempted to improve the rendering model from different perspectives to achieve fast training as well as high-quality real-time rendering, further advancing the development of rendering technology.

Zielonka et al. [92] proposed a Gaussian tiling-based human representation model (D3GA), which enabled real-time rendering through innovative 3DGS techniques. Unlike existing realistic drivable avatars, D3GA does not require precise 3D alignment during training, nor does it require dense input images during testing. Instead, it utilizes densely calibrated multiview video for real-time rendering and introduces a tetrahedral cage-based deformation method driven by keypoints and angles for applications involving communication.

Gaussian injection techniques have also been applied to physical simulations, modeling mechanical properties such as strain through extended parameters. Guedon et al. [90] proposed a method for extracting an accurate mesh from a Gaussian distribution, with improved efficiency through regularization terms and Poisson reconstruction techniques. Duisterhof et al. [93] developed MD-Splatting, combining multi-camera video and neural voxel coding to project Gaussian distributions into metric space, further extending the application of 3DGS.

Gaussian spraying techniques have been extended to 3D editing and point manipulation. Fang et al. [94,95] proposed a Gaussian spraying-based framework for fine 3D scene editing that addressed the limitations of existing diffusion models. Unlike existing methods, that approach achieved precise and localized editing of 3D scenes by exploiting the specific properties of 3D Gaussian functions. These methods not only represent the scene as a 3D Gaussian distribution but also possess a semantic and contextual understanding of the scene, which further enhances the potential of 3DGS in scene editing.

The 3D Gaussian Splatting technique efficiently handles complex geometric and lighting variations through Gaussian functions, demonstrating the potential for a wide range of applications in real-time rendering, dynamic scene reconstruction, SLAM, physics simulation, and scene editing. As the research progresses, the continuous improvement of 3DGS in computational efficiency, rendering quality, and application scope will further advance the fields of computer graphics and computer vision and bring about revolutionary advances in 3D reconstruction and representation techniques in the future.

## 4. Datasets and Evaluation Metrics

### 4.1. Three-Dimensional Reconstruction

#### 4.1.1. Datasets

We summarize the detailed descriptions of commonly used datasets in Table 1.

The TUM RGB-D dataset is a scene dataset captured by Kinect, which contains depth images of RGB images with an image resolution of 640×480. The TUM RGB-D dataset contains 39 sequences and mainly indoor scenes, such as offices, classrooms, and corridors. The correspondence between the RGB images and depth images of this dataset is accurate and the accuracy of the depth data is high.

The KITTI [96] dataset is one of the commonly used datasets in the field of automated driving, which is used to measure the performance of computer vision tasks such as stereo vision, optical flow, 3D object detection, 3D reconstruction, etc., in an in-vehicle environment. The KITTI dataset contains a variety of sensor data, 389 pairs of stereo images and optical flow maps, visual ranging sequences, 3D annotated object images, point cloud data, and truth maps, which are useful for the automatic driving and 3D reconstruction, etc. It provides rich data resources for the fields of automatic driving and 3D reconstruction.

The SUN3D [97] dataset is a real indoor scene dataset released by the Massachusetts Institute of Technology (MIT), which contains 415 different indoor scenes, each of which contains multiple video sequences. The dataset captures indoor environments, including offices, classrooms, conference rooms, living rooms, bedrooms, corridors, and other daily life and working environments through RGB-D sensors, covering different lighting conditions, object layouts, and spatial structures, which provides rich and realistic experimental data for 3D reconstruction.

The ShapeNet [98] dataset is a large-scale, category-rich 3D model database containing 3 million 3D models, whose ShapeNetCore subset contains 220,000 3D models and whose ShapeNetSem subset contains 12,000 models. The ShapeNet dataset is rich in annotation information as well as covering a wide range of object classes. The ShapeNet dataset has rich annotation information and covers a wide range of object classes and scene elements, including furniture, transportation, electronic devices, plants, animals, etc., and can be used in 3D reconstruction techniques based on geometric methods, deep learning, and hybrid methods.

**Figure 10:**
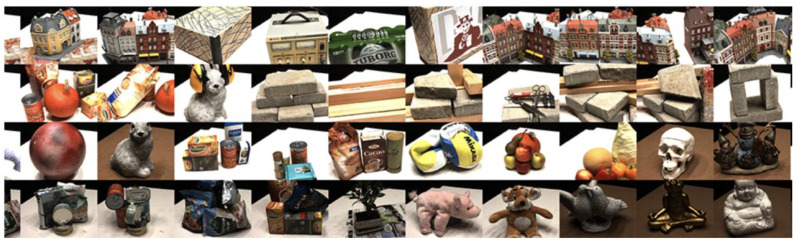
Example image of the DTU dataset.

The ModelNet [99] dataset is a large and diverse benchmark dataset published by the Princeton Vision and Robotics Laboratory, containing 127,915 3D CAD models in 662 categories. The dataset models cover common object categories such as airplanes, cars, chairs, etc. Among them, the ModelNet dataset contains three subsets, ModelNet10 (10 subsets of labeled orientation data), ModelNet40 (40 categories of 3D models), and Aligned40 (40 categories of labeled 3D models). The DTU [100] dataset is a multiview dataset containing 128 different scenes, each containing 49 or 64 views with an image resolution of 1600×1200. These views were all captured by a robot, which precisely controlled the angle and position of the shots to obtain multiview information. In addition, each scene of the DTU dataset has a point cloud of structured light scans in addition to RGB images and depth images; Figure 10 provides an example image of the DTU dataset.

The ETH3D [101] dataset, a dataset proposed by the Swiss Federal Institute of Technology (ETH), contains high-resolution multiview, low-resolution multiview, and low-resolution dual-view data. Among them, there are a total of 35 scenes in high resolution and low resolution, containing many complex indoor and outdoor scenes. Compared with simple datasets, the ETH3D dataset provides more challenges for 3D reconstruction algorithms and better tests the performance and robustness of the algorithms.

The Tanks and Temples [102] dataset is a multiview reconstruction algorithm dataset jointly released by Princeton University and the University of Washington in 2017, which contains 14 kinds of scenes in the Intermediate collection and the Advanced collection. The Intermediate collection contains eight large-scale scenes, such as a family sculpture, an amusement park, and a train. The Advanced collection contains six high-level and detail-rich scenes, such as auditoriums, ballrooms, museums, etc. The Tanks and Temples dataset mainly focuses on high-resolution videos, which can be used for 3D scene reconstruction by capturing images from videos.

The ScanNet [103] dataset is a large-scale RGB-D video dataset containing 1513 indoor scenes. Each scene in this dataset contains a large number of views (about 2.5 million views) and contains scenes of various room types, public areas, and business places, etc. The ScanNet dataset contains RGB-D video, point cloud data, and rich annotation information, which can be used in the fields of 3D reconstruction, target detection, and semantic segmentation.

The Local Light Field Fusion (LLFF) [104] dataset is a dataset of 24 life scenes captured by a cell phone camera, and each scene consists of 20 to 30 images, with image resolutions of 4032×3024, 1008×756, and 504×378, respectively. The LLFF dataset is mainly used for deep learning-based 3D reconstruction and utilizes the light field information to recover the 3D structure of the scene, providing data support of the actual scene for light field 3D reconstruction algorithms.

The University of Texas Multi-modal (UT-MM) [87] dataset is used to evaluate SLAM and 3D reconstruction methods based on multimodal information fusion by providing multiple data modalities such as RGB images, depth images, LiDAR depth, and inertial measurements acquired by a mobile robot. The dataset contains eight scenes with high data quality and accuracy, which can comprehensively reflect the scene information.

#### 4.1.2. Evaluation Metrics

**Intersection over Union Ratio.** Intersection over Union (IoU) is used to measure the difference between the volume of predicted voxels and the volume of real voxels. By calculating the ratio of the intersection and concatenation of the volume of predicted voxels and the volume of real voxels, the accuracy and completeness of the reconstruction results can be intuitively reflected by the calculation of the formula in Equation (5):

(5) $$\mathrm{IOU}=\frac{Prediction\cap GroundTruth}{Prediction\cup GroundTruth}=\frac{\sum_{i,j,k}I\left(P_{\left(i,j,k\right)}-t\right)\cdot I\left(GT_{\left(i,j,k\right)}\right)}{\sum_{i,j,k}\left[I\left(P_{\left(i,j,k\right)}-t\right)+I\left(GT_{\left(i,j,k\right)}\right)\right]}$$

where Prediction denotes the predicted voxel value, GroundTruth denotes the true voxel value. $I\left(\cdot \right)$ denotes the indicator function. $P_{\left(i,j,k\right)}$ and $GT_{\left(i,j,k\right)}$ denote the predicted and true values of the voxels (i,j,k), respectively. *t* represents the voxel threshold. IOU integrates the overlap and overall coverage of the predicted shape and the real shape, and the higher its value, the higher the overlap between the 3D reconstruction result and the real shape, and the better the reconstruction effect.

**F-score.** F-score [102] is an evaluation metric that integrates precision and recall to comprehensively evaluate the results of 3D reconstruction. Precision is mainly used to measure the proportion of the truly correct part of the reconstruction results to the total number of reconstruction results. Recall, on the other hand, measures the proportion of the truly correct part of the reconstruction results to the total number of actual target objects. Therefore, the F-score can find a balance between the two, making the evaluation results more objective; the specific formula is as shown in Equation (6):

(6) $$F\left(d\right)=\frac{2\cdot P(d)\cdot R(d)}{P(d)+R(d)}$$

where P(d) and R(d) represent the precision and recall with respect to the distance threshold.

**Mean Square Error.** The mean square error (MSE) is an averaging of the squares of the differences between the 3D reconstructed shape and the densely sampled points on the real shape and is commonly used to assess the degree of difference between the reconstructed 3D model and the real values, as specified in Equation (7):

(7) $$MSE=\frac{1}{n}\sum_{i=1}^{n}{\left(P_{i}-GT_{i}\right)}^{2}$$

where $P_{i}$ and $GT_{i}$ are the predicted and true values of the *i*th point, respectively. *n* is the total number of points. The smaller the MSE value, the closer the reconstruction result is to the true shape, and the more accurate the reconstruction; on the contrary, a larger MSE means that the reconstruction result is more different from the true shape, and the reconstruction result is less effective.

**Earth Mover’s Distance.** Earth Mover’s Distance (EMD) takes into account the optimal matching between the points and can reflect the difference in overall shape and distribution between the two models, so it can be used to assess the difference in shape between the predicted 3D model and the real model, which is formulated as Equation (8):

(8) $$EMD\left(S_{gt},S_{pre}\right)=\sum_{g\in S_{gt}}∥g-\phi \left(g\right)∥$$

where $S_{gt}$ denotes the set of points of the real model, $S_{pre}$ denotes the set of points of the 3D reconstructed model, $\phi \left(g\right)\in S_{pre}$ is the closest point to $g\in S_{gt}$ in $S_{pre}$. By calculating the distance, point *g* moves to point ϕ(g) according to the movement cost of the point. When the point cloud of the real model is converted to the point cloud of the reconstructed model, the lower the “transportation cost” required, i.e., the more similar the distributions of the two point clouds, the more similarity between the models, the better the reconstruction effect; on the contrary, the bigger the value of EMD, the worse the reconstruction effect.

**Chamfer Distance.** The Chamfer Distance (CD) is a distance metric used to measure the similarity between two point clouds or shapes, reflecting the overall degree of similarity between the two point clouds. The CD essentially calculates the sum of the distances from each point in a point cloud to the nearest neighboring point in the other point cloud and adds the sum of the distances of the two point clouds; the smaller the distance, the more similar the two point clouds, as specified by the formula Equation (9):

(9) $$d_{CD}\left(S_{1},S_{2}\right)=\frac{1}{S_{1}}\sum_{x\in S_{1}}\lim_{y\in S_{2}}{∥x-y∥}_{2}^{2}+\frac{1}{S_{1}}\sum_{y\in S_{2}}\lim_{x\in S_{1}}{∥y-x∥}_{2}^{2}$$

where $S_{1}$ and $S_{2}$ denote two sets of 3D point clouds, respectively, and the first term represents the sum of the minimum distances from any point $x\in S_{1}$ to $S_{2}$, and the second term represents the sum of the minimum distances from any point $y\in S_{2}$ to $S_{1}$. If the CD is large, it means that the difference between the two 3D point clouds is large.

**Completeness Ratio.** The Completeness Ratio (CR) is one of the metrics used to evaluate the quality of 3D reconstruction and to measure the degree of completeness of the information contained in the reconstructed 3D model with respect to the information of the real model. It is usually used to determine the degree of coverage of the reconstructed model over the real model by comparing the reconstructed model with the real model in terms of specific features, such as the number of point clouds, the surface area, the number of voxels, and so on. The specific formula for the completeness ratio is in Equation (10):

(10) $$CR=\frac{\left|P\cap GT\right|}{\left|GT\right|}$$

where |P∩GT| denotes the number of points where the 3D-reconstructed point cloud overlaps with the real point cloud, and GT indicates the total number of points in the real point cloud. The closer the value of the integrity ratio is to one, the higher the reconstruction quality is; on the contrary, the lower the ratio, the more missing information the reconstruction result has, and the poorer the reconstruction quality.

### 4.2. Three-Dimensional Rendering

#### 4.2.1. Datasets

In recent years, 3DGS has been rapidly growing in the field of computer graphics due to its advantages of an efficient and high-quality rendering of 3D scenes. However, most datasets focus mainly on 3D reconstruction tasks, and the number of datasets constructed specifically for rendering models is limited. For this reason, this paper investigated and summarized 3D reconstruction rendering methods and generalized datasets suitable for 3DGS rendering models.

The Tanks and Temples [102] dataset contains high-definition video clips of indoor and outdoor environments as well as complex lighting conditions, which can comprehensively test the rendering ability of the model in different scenes, especially the preservation of details and the rendering ability of the overall effect when dealing with large and complex scenes.

The DeepBlending [105] dataset collects a total of 2630 images from different sources for 19 scenes, including 5 indoor scenes and 5 scenes with a lot of vegetation, each containing 12 to 418 input images with image resolutions ranging from 1228×816 to 2592×1944. In addition, this dataset is augmented with data to increase the diversity of the data as well as to reduce the risk of over-cropping, with 90% of the images in each scene being the training set and the remaining 10% serving as the validation set.

**Figure 11:**
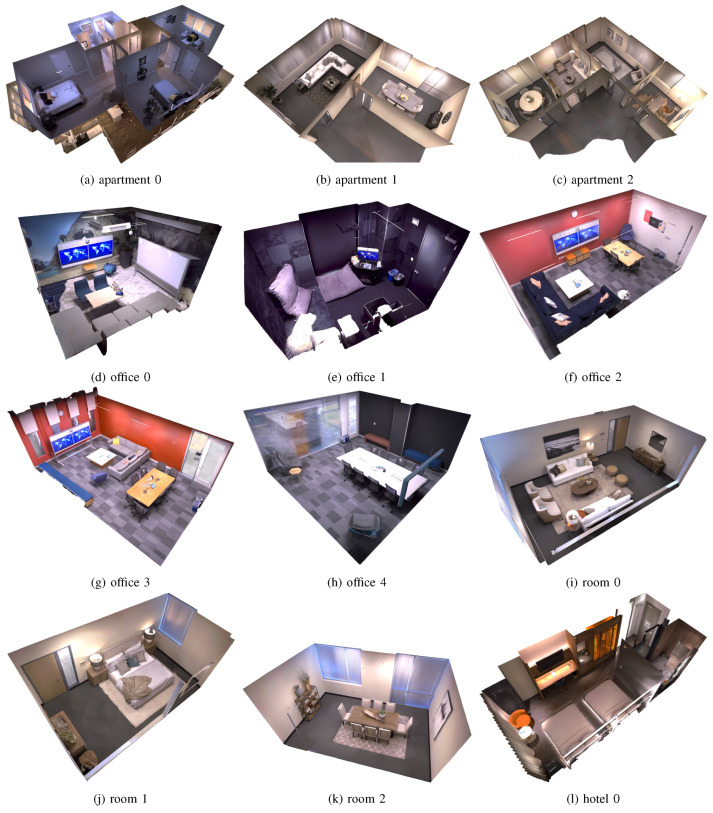
The Replica dataset contains 12 semantically distinct reconstruction examples [7].

The Replica [106] dataset is a large-scale indoor scene reconstruction dataset, where 18 different scenes were captured by using a customized RGB-D device, each featuring a dense mesh, high-resolution High Dynamic Range (HDR) textures, semantic data, and reflective surfaces to maintain visual realism. This dataset has rich scene features and representations to improve the rendering and generalization of the model, and an example reconstruction of this dataset is shown in Figure 11.

The BlendedMVS [107] dataset is a large synthetic dataset for multiview stereo (MVS) matching training. The dataset contains a total of 113 scenes, each containing between 20 and 1000 input images, for a total of 17,818 high-resolution images covering a wide variety of landscapes such as urban areas, structures, sculptures, and micro-objects, which provide a large number of training examples for MVS studies.

The Mip-NeRF 360 [108] dataset was constructed primarily for the challenge of unbounded scenes (camera facing any direction, content distributed at any distance). The data capture multiview images with depth information for a total of nine scenes (five outdoor and four indoor) with an image resolution of 1008×756 pixels, which can be used to evaluate the model’s ability to generate realistic views and accurate depth maps.

The BungeeNeRF [109] dataset is a multi-scale scene dataset used for training and evaluating the BungeeNeRF model and contains RGB images at different scales. Twelve city (e.g., New York, Chicago, Sydney) scenes were acquired by using Google Earth Studio, from satellite level to ground level, and each scene contains image data at different scales. The BungeeNeRF dataset can help the model to deal with multi-scale scene-rendering problems effectively, and it performs especially well in near-field details and far-field completeness.

UrbanScene3D [110] is a large-scale dataset for studying urban scene perception and reconstruction, containing 128,000 high-resolution images covering 16 scenes, including large-scale real urban areas and synthetic cities with a total area of 136 square kilometers. The dataset is rich in data types, including depth maps, 3D point clouds, and LIDAR scans, which can help models learn and process diverse urban scenes to produce more realistic and accurate rendering results.

The TensoIR [111] dataset was generated by Blender software rendering and constructed for evaluating the TensoIR model. The dataset contains four complex composite scenes, ficus, lego, armadillo and hot dog, with 100 training views and 200 test views for each scene, and each view is provided with normal vector maps, albedo maps, and 11 RGB images under different lighting. This dataset can accurately model scene geometry, albedo, and illumination and can be used to accurately model shadows and indirect illumination using information from multiple lighting conditions.

#### 4.2.2. Evaluation Metrics

**Peak Signal-to-Noise Ratio.** The Peak Signal-to-Noise Ratio (PSNR) [112] is an objective metric used to measure the degree of distortion or quality of an image by calculating the difference in pixel values between the original image and the processed image in order to assess the image quality, which is calculated as in Equation (11):

(11) $$PSNR=10\cdot log_{10}\left(\frac{MAX^{2}}{MSE}\right)$$

where MSE is the mean square error and MAX is the maximum value of the original image pixel value. A higher value of PSNR indicates higher image quality; conversely, a lower value indicates poor image quality.

**Structural Similarity.** Structural similarity (SSIM) [109] evaluates the image quality from the perspective of the structural similarity of the image and measures the image quality by comparing the similarity between the original image and the processed image in the three aspects of luminance, contrast, and structure, which are given by the formula Equation (12):

(12) $$SSIM\left(x,y\right)=\frac{\left(2\mu_{x}\mu_{y}+C_{1}\right)\left(2\sigma_{xy}+C_{2}\right)}{\left(2\mu_{x}^{2}\mu_{y}^{2}+C_{1}\right)\left(\sigma_{x}^{2}+\sigma_{y}^{2}+C_{2}\right)}$$

where *x* and *y* are the original image and the processed image, respectively. $\mu_{x}$ and $\mu_{y}$ are the mean of *x* and *y*, respectively. $\sigma_{x}$ and $\sigma_{y}$ are the standard deviation of *x* and *y*, respectively. $\sigma_{xy}$ is the covariance of *x* and *y*, and $C_{1}$ and $C_{2}$ are constants to avoid a computationally unstable situation. The results of SSIM are usually in the range of −1 to 1, with values closer to 1 indicating greater similarity between the two images, and values closer to −1 indicating a larger difference.

**Learned Perceptual Image Patch Similarity.** Learned Perceptual Image Patch Similarity (LPIPS) [109], also known as Perceptual Loss, is a measure of the perceptual difference between the original image and the processed image.

LPIPS is more in line with the human visual judgment of image quality by using convolutional neural networks for local feature extraction of the image and calculating the similarity between patches. After computing patch distances across different convolutional layers, the LPIPS value is obtained by a weighted sum of these distances, synthesizing similarity information at various levels of the feature space. A smaller LPIPS value indicates greater perceptual similarity between the reference and distorted images, while a larger value suggests greater perceptual difference. In recent studies, these metrics have been widely used to evaluate the quality of 3D rendering and reconstruction results. For instance, Kerbl et al. [9] employed LPIPS, along with PSNR and SSIM, to quantitatively assess the perceptual quality of images generated by their 3D Gaussian Splatting framework. The use of LPIPS in their evaluation highlighted its effectiveness in capturing perceptual differences that are often not reflected by traditional pixel-wise metrics.

To better illustrate the comparative performance of representative 3D reconstruction and rendering methods, we summarize key metrics including PSNR, SSIM, LPIPS, and rendering speed in Table 2, based on benchmark datasets and published evaluations. Table 2 presents a performance comparison of mainstream 3D reconstruction and rendering methods and their variants in recent years. For each variant, we specify the exact method name in parentheses. For example, 3DGS (GsPro) refers to a variant of the 3DGS method called GsPro. We can observe that 3DGS and its variants offer significant advantages in rendering speed while maintaining high image quality, making them more suitable for practical deployment and real-time scenarios. In contrast, although NeRF and its improved versions perform well in terms of image detail, they suffer from clear bottlenecks in rendering speed.

## 5. Applications

With advanced algorithms, model architectures, and innovative applications of deep learning, 3D scene reconstruction and rendering technology breaks through the limitations of traditional 2D images to achieve accurate 3D modeling and high-quality rendering of objects and scenes. In Table 3, we further summarize the strengths and limitations of different methods across representative application domains, providing guidance for method selection in practice. The following introduces the applications of 3D reconstruction and rendering technology in different fields one by one.

In cultural heritage protection and virtual museum construction, 3D reconstruction helps to restore and preserve historical sites and artifacts, while rendering technology transforms these models into highly realistic virtual images or animations, enabling users to explore immersive digital spaces. For example, NeuroVerse [7] demonstrated this integration by reconstructing a historical building of the University of Turin and embedding it into a virtual environment for collaborative scientific analysis and education. Similarly, in architectural design and urban planning, designers can simulate and evaluate design schemes in virtual environments through 3D modeling, supported by rendering techniques that produce realistic visualizations. In the fields of VR games, film and television production, and animation, 3D reconstruction technology can create high-quality 3D models for the virtual world, and rendering technology can generate realistic images that conform to the laws of light and shadow. In medical imaging and surgical planning, 3D reconstruction of CT scans, MRIs, and other medical images can accurately render a patient’s anatomy. Rendering technology is used to transform these medical models into visualized images. By combining virtual reality technology with surgical simulation, surgical risks can be reduced, and surgical precision can be improved. In addition, in the field of autonomous driving, 3D scene reconstruction technology is used to obtain 3D data of the vehicle’s surroundings, and rendering technology is used to generate high-quality images of the environment, which can help the system perform scene understanding, target detection, and path planning.

The application of 3D scene reconstruction and rendering technologies spans a wide range of fields, showing their great value in both the real and virtual worlds. With the continuous progress of technology, the application of 3D scene reconstruction and rendering will be further deepened in the future, bringing richer application scenarios and great social benefits.

## 6. Challenges and Future Trends

Despite the rapid advancement of 3D reconstruction and rendering techniques, several challenges remain. Beyond issues specific to deep learning methods, such as data dependency, computational complexity, and generalization, there are broader concerns related to dataset quality, scalability of models, and integration of neural techniques with traditional rendering pipelines. In addition, real-time performance, and robustness in dynamic or large-scale environments continue to pose limitations for practical deployment.

Looking ahead, we identify several promising directions. The development of hybrid frameworks that combine traditional geometric priors with learning-based flexibility may offer better generalization and interpretability. Lightweight and energy-efficient models will be critical for deployment on mobile or edge devices. Moreover, the use of large-scale synthetic datasets, self-supervised learning, and generative techniques such as GANs or diffusion models is expected to reduce reliance on labeled data and improve reconstruction quality. In particular, 3DGS shows strong potential in areas such as immersive content creation, virtual reality applications, and real-time AR/VR rendering, indicating that it will play an increasingly important role in the future of 3D vision.

## 7. Conclusions

This study briefly reviewed the development of 3D Gaussian Splatting (3DGS), a revolutionary technique that greatly accelerates the rendering process in novel view synthesis by virtue of its unique mathematical model and algorithmic advantages. Its core lies in the direct estimation of 3D shapes using Gaussian distributions, which has attracted the attention of scholars and engineers at home and abroad and has gradually reshaped the development paths of various fields such as explicit radiation fields, computer graphics, and computer vision. In this paper, we focused on 3D scene reconstruction and rendering, analyzed in detail the basic principle and implementation mechanism of 3DGS, and demonstrated its excellent performance in practical applications through a large number of literature studies and theoretical analyses. We not only summarized the current breakthroughs of 3DGS technology in data processing, real-time rendering, and accuracy enhancement but also revealed its challenges in handling large-scale dynamic scenes and maintaining spatio-temporal consistency. By comparing the advantages and disadvantages of traditional methods and 3DGS technology, this paper proposed future improvement directions, including multi-sensor data fusion, deep learning-assisted optimization, and the integrated application of fine-grained structure optimization algorithms. The 3DGS technology, as a far-reaching innovative technology, is expected to significantly promote the development of 3D reconstruction and rendering technology in the future and provide more accurate and efficient technological support for the fields of virtual reality, augmented reality, and intelligent manufacturing. This survey aimed to provide fundamental resources and theoretical guidance for further research in this field and to promote interdisciplinary cooperation and technological progress.

## Figures and Tables

**Table 1:** Commonly used 3D reconstruction datasets.

Year	Dataset	Data Composition	Description	Tasks
2012	TUM RGB-D	39 sequences: RGB/depth images, camera pose	Indoor scenes from RGB-D camera	Quantitative evaluation
2013	KITTI	14,999 images: RGB/grayscale images, point clouds, GPS/IMU/calibration data, annotations	Large-scale outdoor scenes from cameras, Velodyne HDL-64E LiDAR and GPS/IMU	Recognition, segmentation, and optical flow estimation
2013	SUN3D	415 scenes: depth images, point clouds, camera poses, semantic segmentation	Multiview indoor scenes from RGB-D sensors	Reconstruction and understanding
2015	ShapeNet	3M models: 3D CAD models	3D model categories	Recognition, generation, and classification
2015	ModelNet	127,915 models: 3D CAD models	3D model categories	Classification
2016	DTU	128 scenes: RGB/depth images, point clouds, camera parameter	Multiview stereo depth indoor dataset from industrial robot	Reconstruction, depth estimation, and visual SLAM
2017	ETH3D	35 scenes: RGB images	Indoor/out scenes from DSLR cameras	Depth estimation and reconstruction
2017	Tanks and Temples	14 scenes: point clouds, video sequences	Indoor/outdoor 3D reconstruction dataset from cameras and LiDAR	Reconstruction
2017	ScanNet	1513 scenes: RGB-D videos, point clouds	Indoor/outdoor scenes with semantic images from RGB-D cameras	Reconstruction, recognition, and understanding
2019	LLFF	24 scenes: RGB images, pose estimation data	Light field indoor data from multiview smartphone cameras	View synthesis and reconstruction
2022	Mip-NeRF 360	9 scenes including 5 outdoor and 4 indoor	Multiview images with depth information with resolution of 1008×756 pixels	Unbounded scenes (camera facing any direction, content distributed at any distance)
2024	UT-MM	8 scenes: RGB/depth images, LiDAR, IMU, etc.	Multimodal indoor/outdoor scenes from mobile robot	Multimodal learning and cross-modal retrieval

**Table 2:** Comparison of quantitative performance of mainstream methods on standardized datasets, assessed by PSNR (higher is better ), SSIM (higher is better), LPIPS (lower is better), FPS (higher is better).

Methods	Datasets	PSNR ↑	SSIM ↑	LPIPS ↓	FPS ↑
3DGS [9]	Tanks and Temples	23.14	0.841	0.183	154
3DGS (OPS) [113]	Tanks and Temples	26.44	0.872	0.214	–
NeRF (Mip-NeRF 360) [108]	Tanks and Temples	22.22	0.760	0.260	0.14
3DGS [9]	Mip-NeRF 360	27.21	0.815	0.214	134
3DGS (OPS) [113]	Mip-NeRF 360	27.48	0.821	0.209	–
3DGS (Compact3D) [114]	Mip-NeRF 360	27.16	0.808	0.228	–
3DGS (EAGLES) [115]	Mip-NeRF 360	27.15	0.810	0.240	–
3DGS (GsPro) [116]	Mip-NeRF 360	27.92	0.825	0.208	–
3DGS (C3DGS) [117]	Mip-NeRF 360	27.08	0.798	0.247	–
NeRF (Mip-NeRF 360) [108]	Mip-NeRF 360	27.69	0.790	0.240	0.06
3DGS [9]	LLFF	24.37	0.822	0.262	445
NeRF [7]	LLFF	26.50	0.811	0.250	–
NeRF (Super-NeRF) [118]	LLFF	27.75	0.863	0.087	–
3DGS (MPGS) [119]	LLFF	27.15	0.868	0.202	469
3DGS (GSPro) [116]	LLFF	26.83	0.869	0.196	199
3DGS (SurfelGS) [120]	LLFF	26.73	0.869	0.190	107
3DGS (GoF) [121]	LLFF	26.93	0.867	0.182	388

**Table 3:** Summary of the ability of each mainstream method to adapt to different application scenarios.

Application Domain	Representative Methods	Strengths	Challenges
Real-Time Rendering and View Synthesis	3DGS [9], EAGLES [115], MPGS [119]	Real-time performance, high-quality novel view synthesis, compatible with rasterization pipelines	Multi-scale rendering artifacts, high memory consumption
Autonomous Driving and Dynamic Scenes	GaussianPro [116], C3DGS [117], NeRF [7]	Dynamic object modeling, real-time LiDAR data fusion, strong cross-dataset generalization	Difficulty handling motion blur, high computational load in large-scale scenes
Surface Reconstruction and Mesh Generation	SurfelGS [120], OPS [113]	High-fidelity surface details, adaptability to unbounded scenes, compatibility with traditional mesh pipelines	Instability in transparent/reflective object reconstruction, requires post-processing mesh optimization
High-Resolution and Anti-Aliased Rendering	Super-NeRF [118], Mip-NeRF 360 [108]	View-consistent detail generation at high resolution, anti-aliasing for unbounded scenes, continuous level-of-detail representation	High computational cost, limited real-time capability
Compression and Efficient Representation	Compact3D [114], MPGS [119]	High compression ratio, maintains real-time rendering performance, supports multi-resolution streaming	Quantization error accumulation, loss of high-frequency details
VR/AR and Interactive Editing	NeRF [7], GoF [121]	User-controllable deformations, real-time interactive modifications	Lag in shadow/lighting updates, difficulty maintaining geometric consistency after edits

## References

[1] Schonberger J.L., Frahm J.M. Structure-from-motion revisited. Proceedings of the 2016 IEEE Conference on Computer Vision and Pattern Recognition (CVPR).

[2] Zwicker M., Pfister H., Van Baar J., Gross M. Surface splatting. Proceedings of the 28th Annual Conference on Computer Graphics and Interactive Techniques.

[3] Kopanas G., Philip J., Leimkühler T., Drettakis G. (2021). Point-Based Neural Rendering with Per-View Optimization. Computer Graphics Forum.

[4] Zwicker M., Pfister H., Van Baar J., Gross M. (2001). EWA volume splatting. Proceedings of the Proceedings Visualization, 2001. VIS ’01.

[5] Botsch M., Kobbelt L. (2003). High-quality point-based rendering on modern GPUs. Proceedings of the 11th Pacific Conference on Computer Graphics and Applications.

[6] Rusinkiewicz S., Levoy M. QSplat: A multiresolution point rendering system for large meshes. Proceedings of the 27th Annual Conference on Computer Graphics and Interactive Techniques.

[7] Mildenhall B., Srinivasan P.P., Tancik M., Barron J.T., Ramamoorthi R., Ng R. (2021). Nerf: Representing scenes as neural radiance fields for view synthesis. Commun. ACM.

[8] Yifan W., Serena F., Wu S., Öztireli C., Sorkine-Hornung O. (2019). Differentiable surface splatting for point-based geometry processing. ACM Trans. Graph. (TOG).

[9] Kerbl B., Kopanas G., Leimkühler T., Drettakis G. (2023). 3D gaussian splatting for real-time radiance field rendering. ACM Trans. Graph..

[10] Horn B.K.P. (1970). Shape from Shading: A Method for Obtaining the Shape of a smooth Opaque Object from One View.

[11] Fan J., Chen M., Mo J., Wang S., Liang Q. (2022). Variational formulation of a hybrid perspective shape from shading model. Vis. Comput..

[12] Woodham R.J. (1980). Photometric method for determining surface orientation from multiple images. Opt. Eng..

[13] Shi B., Matsushita Y., Wei Y., Xu C., Tan P. (2010). Self-calibrating photometric stereo. Proceedings of the 2010 IEEE Computer Society Conference on Computer Vision and Pattern Recognition.

[14] Morris N.J., Kutulakos K.N. (2011). Dynamic refraction stereo. IEEE Trans. Pattern Anal. Mach. Intell..

[15] Shen H.L., Han T.Q., Li C. (2016). Efficient photometric stereo using kernel regression. IEEE Trans. Image Process..

[16] Sturm P., Triggs B. (1996). A factorization based algorithm for multi-image projective structure and motion. Proceedings of the Computer Vision—ECCV’96: 4th European Conference on Computer Vision.

[17] Crandall D., Owens A., Snavely N., Huttenlocher D. (2011). Discrete-continuous optimization for large-scale structure from motion. Proceedings of the CVPR 2011.

[18] Moulon P., Monasse P., Marlet R. Global fusion of relative motions for robust, accurate and scalable structure from motion. Proceedings of the 2013 IEEE International Conference on Computer Vision.

[19] Zhu S., Zhang R., Zhou L., Shen T., Fang T., Tan P., Quan L. Very large-scale global sfm by distributed motion averaging. Proceedings of the 2018 IEEE/CVF Conference on Computer Vision and Pattern Recognition.

[20] Wu C. (2013). Towards linear-time incremental structure from motion. Proceedings of the 2013 International Conference on 3D Vision—3DV 2013.

[21] Wang J., Zhong Y., Dai Y., Birchfield S., Zhang K., Smolyanskiy N., Li H. Deep two-view structure-from-motion revisited. Proceedings of the 2021 IEEE/CVF Conference on Computer Vision and Pattern Recognition (CVPR).

[22] Qu Y., Huang J., Zhang X. (2018). Rapid 3D reconstruction for image sequence acquired from UAV camera. Sensors.

[23] Cui H., Shen S., Gao W., Liu H., Wang Z. (2019). Efficient and robust large-scale structure-from-motion via track selection and camera prioritization. Isprs J. Photogramm. Remote. Sens..

[24] Cui H., Gao X., Shen S., Hu Z. HSfM: Hybrid structure-from-motion. Proceedings of the 2017 IEEE Conference on Computer Vision and Pattern Recognition (CVPR).

[25] Zhu S., Shen T., Zhou L., Zhang R., Wang J., Fang T., Quan L. (2017). Parallel structure from motion from local increment to global averaging. arXiv.

[26] Scharstein D., Szeliski R. (2002). A taxonomy and evaluation of dense two-frame stereo correspondence algorithms. Int. J. Comput. Vis..

[27] Huang L., Wu G., Liu J., Yang S., Cao Q., Ding W., Tang W. (2020). Obstacle distance measurement based on binocular vision for high-voltage transmission lines using a cable inspection robot. Sci. Prog..

[28] Wei M., Shoulei M., Jianjian B., Chenbo Y., Donghui C., Hongfu Y. (2020). Three-dimensional Reconstruction of Working Environment in Remote Control Excavator. Proceedings of the 2020 5th International Conference on Mechanical, Control and Computer Engineering (ICMCCE).

[29] Xu T., Chen Z., Jiang Z., Huang J., Gui W. (2020). A real-time 3D measurement system for the blast furnace burden surface using high-temperature industrial endoscope. Sensors.

[30] Furukawa Y., Hernández C. (2015). Multi-View Stereo: A Tutorial.

[31] Furukawa Y., Ponce J. (2009). Accurate, dense, and robust multiview stereopsis. IEEE Trans. Pattern Anal. Mach. Intell..

[32] Shen S. (2013). Accurate multiple view 3D reconstruction using patch-based stereo for large-scale scenes. IEEE Trans. Image Process..

[33] Zheng E., Dunn E., Jojic V., Frahm J.M. Patchmatch based joint view selection and depthmap estimation. Proceedings of the 2014 IEEE Conference on Computer Vision and Pattern Recognition.

[34] Jashari S., Tukur M., Boraey Y., Alzubaidi M., Pintore G., Gobbetti E., Villanueva A.J., Schneider J., Fetais N., Agus M. Evaluating AI-based static stereoscopic rendering of indoor panoramic scenes. Proceedings of the STAG: Smart Tools and Applications in Graphics (2024).

[35] Pintore G., Jaspe-Villanueva A., Hadwiger M., Gobbetti E., Schneider J., Agus M. PanoVerse: Automatic generation of stereoscopic environments from single indoor panoramic images for Metaverse applications. Proceedings of the 28th International ACM Conference on 3D Web Technology.

[36] Gunatilake A., Piyathilaka L., Kodagoda S., Barclay S., Vitanage D. (2019). Real-time 3D profiling with RGB-D mapping in pipelines using stereo camera vision and structured IR laser ring. Proceedings of the 2019 14th IEEE Conference on Industrial Electronics and Applications (ICIEA).

[37] Montusiewicz J., Miłosz M., Kęsik J., Żyła K. (2021). Structured-light 3D scanning of exhibited historical clothing—A first-ever methodical trial and its results. Herit. Sci..

[38] Chen X., Li Q., Wang T., Xue T., Pang J. Gennbv: Generalizable next-best-view policy for active 3D reconstruction. Proceedings of the 2024 IEEE/CVF Conference on Computer Vision and Pattern Recognition (CVPR).

[39] Guðmundsson S.Á., Pardàs M., Casas J.R., Sveinsson J.R., Aanæs H., Larsen R. (2010). Improved 3D reconstruction in smart-room environments using ToF imaging. Comput. Vis. Image Underst..

[40] Hoegg T., Lefloch D., Kolb A. (2013). Time-of-Flight camera based 3D point cloud reconstruction of a car. Comput. Ind..

[41] Li L., Liu H., Xu Y., Zheng Y. (2020). Measurement linearity and accuracy optimization for time-of-flight range imaging cameras. Proceedings of the 2020 IEEE 4th Information Technology, Networking, Electronic and Automation Control Conference (ITNEC).

[42] Fitrah P.A., Ramadhani C.R., Rahmi D.A., Harisna N. (2024). Use of LiDAR in Topographic Map Mapping or Surface Mapping. J. Front. Res. Sci. Eng..

[43] Liao G., Li J., Ye X. VLM2Scene: Self-supervised image-text-LiDAR learning with foundation models for autonomous driving scene understanding. Proceedings of the AAAI Conference on Artificial Intelligence.

[44] Schwalbe E., Maas H.G., Seidel F. (2005). 3D building model generation from airborne laser scanner data using 2D GIS data and orthogonal point cloud projections. Proc. ISPRS WG III/3 III/4.

[45] Feng H., Chen Y., Luo Z., Sun W., Li W., Li J. (2022). Automated extraction of building instances from dual-channel airborne LiDAR point clouds. Int. J. Appl. Earth Obs. Geoinf..

[46] Zhong X., Pan Y., Stachniss C., Behley J. 3D LiDAR Mapping in Dynamic Environments Using a 4D Implicit Neural Representation. Proceedings of the 2024 IEEE/CVF Conference on Computer Vision and Pattern Recognition (CVPR).

[47] Tukur M., Boraey Y., Jashari S., Villanueva A.J., Shah U., Al-Zubaidi M., Pintore G., Gobbetti E., Schneider J., Agus M. (2024). Virtual Staging Technologies for the Metaverse. Proceedings of the 2024 2nd International Conference on Intelligent Metaverse Technologies & Applications (iMETA).

[48] Seitz S.M., Curless B., Diebel J., Scharstein D., Szeliski R. (2006). A comparison and evaluation of multi-view stereo reconstruction algorithms. Proceedings of the 2006 IEEE Computer Society Conference on Computer Vision and Pattern Recognition (CVPR’06).

[49] Yao Y., Luo Z., Li S., Fang T., Quan L. Mvsnet: Depth inference for unstructured multi-view stereo. Proceedings of the European Conference on Computer Vision (ECCV).

[50] Chen R., Han S., Xu J., Su H. Point-based multi-view stereo network. Proceedings of the 2019 IEEE/CVF International Conference on Computer Vision (ICCV).

[51] Huang B., Yi H., Huang C., He Y., Liu J., Liu X. (2021). M3VSNet: Unsupervised multi-metric multi-view stereo network. Proceedings of the 2021 IEEE International Conference on Image Processing (ICIP).

[52] Nichol A., Jun H., Dhariwal P., Mishkin P., Chen M. (2022). Point-e: A system for generating 3D point clouds from complex prompts. arXiv.

[53] Li S., Gao P., Tan X., Wei M. Proxyformer: Proxy alignment assisted point cloud completion with missing part sensitive transformer. Proceedings of the 2023 IEEE/CVF Conference on Computer Vision and Pattern Recognition (CVPR).

[54] Zhang Y., Zhu J., Lin L. Multi-view stereo representation revist: Region-aware mvsnet. Proceedings of the 2023 IEEE/CVF Conference on Computer Vision and Pattern Recognition (CVPR).

[55] Koch S., Hermosilla P., Vaskevicius N., Colosi M., Ropinski T. Sgrec3d: Self-supervised 3D scene graph learning via object-level scene reconstruction. Proceedings of the 2024 IEEE/CVF Winter Conference on Applications of Computer Vision (WACV).

[56] Ji M., Gall J., Zheng H., Liu Y., Fang L. Surfacenet: An end-to-end 3D neural network for multiview stereopsis. Proceedings of the 2017 IEEE International Conference on Computer Vision (ICCV).

[57] Kar A., Häne C., Malik J. (2017). Learning a multi-view stereo machine. Adv. Neural Inf. Process. Syst..

[58] Liu S., Giles L., Ororbia A. (2018). Learning a hierarchical latent-variable model of 3D shapes. Proceedings of the 2018 International Conference on 3D Vision (3DV).

[59] Wang D., Cui X., Chen X., Zou Z., Shi T., Salcudean S., Wang Z.J., Ward R. Multi-view 3D reconstruction with transformers. Proceedings of the 2021 IEEE/CVF International Conference on Computer Vision (ICCV).

[60] Schwarz K., Sauer A., Niemeyer M., Liao Y., Geiger A. (2022). Voxgraf: Fast 3D-aware image synthesis with sparse voxel grids. Adv. Neural Inf. Process. Syst..

[61] Wang Y., Guan T., Chen Z., Luo Y., Luo K., Ju L. Mesh-guided multi-view stereo with pyramid architecture. Proceedings of the 2020 IEEE/CVF Conference on Computer Vision and Pattern Recognition (CVPR).

[62] Yuan Y., Tang J., Zou Z. (2021). Vanet: A view attention guided network for 3D reconstruction from single and multi-view images. Proceedings of the 2021 IEEE International Conference on Multimedia and Expo (ICME).

[63] Ju J., Tseng C.W., Bailo O., Dikov G., Ghafoorian M. Dg-recon: Depth-guided neural 3d scene reconstruction. Proceedings of the 2023 IEEE/CVF International Conference on Computer Vision (ICCV).

[64] Wang Z., Zhou S., Park J.J., Paschalidou D., You S., Wetzstein G., Guibas L., Kadambi A. Alto: Alternating latent topologies for implicit 3D reconstruction. Proceedings of the 2023 IEEE/CVF Conference on Computer Vision and Pattern Recognition (CVPR).

[65] Dogaru A., Özer M., Egger B. (2024). Generalizable 3D scene reconstruction via divide and conquer from a single view. arXiv.

[66] Huang P.H., Matzen K., Kopf J., Ahuja N., Huang J.B. Deepmvs: Learning multi-view stereopsis. Proceedings of the 2018 IEEE/CVF Conference on Computer Vision and Pattern Recognition.

[67] Yi H., Wei Z., Ding M., Zhang R., Chen Y., Wang G., Tai Y.W. (2020). Pyramid multi-view stereo net with self-adaptive view aggregation. Proceedings of the Computer Vision–ECCV 2020: 16th European Conference.

[68] Zhang Z., Peng R., Hu Y., Wang R. Geomvsnet: Learning multi-view stereo with geometry perception. Proceedings of the 2023 IEEE/CVF Conference on Computer Vision and Pattern Recognition (CVPR).

[69] Dib A., Bharaj G., Ahn J., Thébault C., Gosselin P., Romeo M., Chevallier L. (2021). Practical face reconstruction via differentiable ray tracing. Computer Graphics Forum.

[70] Zhang Y., Orth A., England D., Sussman B. (2022). Ray tracing with quantum correlated photons to image a three-dimensional scene. Phys. Rev. A.

[71] Ma J., Liu M., Ahmedt-Aristizabal D., Nguyen C. HashPoint: Accelerated Point Searching and Sampling for Neural Rendering. Proceedings of the 2024 IEEE/CVF Conference on Computer Vision and Pattern Recognition (CVPR).

[72] Anciukevičius T., Xu Z., Fisher M., Henderson P., Bilen H., Mitra N.J., Guerrero P. Renderdiffusion: Image diffusion for 3D reconstruction, inpainting and generation. Proceedings of the 2023 IEEE/CVF Conference on Computer Vision and Pattern Recognition (CVPR).

[73] Zhou Z., Tulsiani S. Sparsefusion: Distilling view-conditioned diffusion for 3D reconstruction. Proceedings of the 2023 IEEE/CVF Conference on Computer Vision and Pattern Recognition (CVPR).

[74] An S., Xu H., Shi Y., Song G., Ogras U.Y., Luo L. Panohead: Geometry-aware 3D full-head synthesis in 360deg. Proceedings of the 2023 IEEE/CVF Conference on Computer Vision and Pattern Recognition (CVPR).

[75] Mirzaei A., Aumentado-Armstrong T., Derpanis K.G., Kelly J., Brubaker M.A., Gilitschenski I., Levinshtein A. Spin-nerf: Multiview segmentation and perceptual inpainting with neural radiance fields. Proceedings of the 2023 IEEE/CVF Conference on Computer Vision and Pattern Recognition (CVPR).

[76] Bian W., Wang Z., Li K., Bian J.W., Prisacariu V.A. Nope-nerf: Optimising neural radiance field with no pose prior. Proceedings of the 2023 IEEE/CVF Conference on Computer Vision and Pattern Recognition (CVPR).

[77] Wynn J., Turmukhambetov D. Diffusionerf: Regularizing neural radiance fields with denoising diffusion models. Proceedings of the 2023 IEEE/CVF Conference on Computer Vision and Pattern Recognition (CVPR).

[78] Wu G., Yi T., Fang J., Xie L., Zhang X., Wei W., Liu W., Tian Q., Wang X. 4d gaussian splatting for real-time dynamic scene rendering. Proceedings of the 2024 IEEE/CVF Conference on Computer Vision and Pattern Recognition (CVPR).

[79] Sless L., El Shlomo B., Cohen G., Oron S. Road scene understanding by occupancy grid learning from sparse radar clusters using semantic segmentation. Proceedings of the 2019 IEEE/CVF International Conference on Computer Vision Workshop (ICCVW).

[80] Zhao P., Lu C.X., Wang J., Chen C., Wang W., Trigoni N., Markham A. (2021). Human tracking and identification through a millimeter wave radar. Ad. Hoc. Networks.

[81] Zhang K., Bi S., Tan H., Xiangli Y., Zhao N., Sunkavalli K., Xu Z. (2024). Gs-lrm: Large reconstruction model for 3D gaussian splatting. European Conference on Computer Vision.

[82] Chen C., Fragonara L.Z., Tsourdos A. (2021). RoIFusion: 3D object detection from LiDAR and vision. IEEE Access.

[83] Gao J., Gu C., Lin Y., Li Z., Zhu H., Cao X., Zhang L., Yao Y. (2024). Relightable 3D gaussians: Realistic point cloud relighting with brdf decomposition and ray tracing. European Conference on Computer Vision.

[84] Lee B., Lee H., Sun X., Ali U., Park E. (2024). Deblurring 3D gaussian splatting. European Conference on Computer Vision.

[85] Liu Y., Luo C., Fan L., Wang N., Peng J., Zhang Z. (2024). Citygaussian: Real-time high-quality large-scale scene rendering with gaussians. European Conference on Computer Vision.

[86] Yu Z., Chen A., Huang B., Sattler T., Geiger A. Mip-splatting: Alias-free 3D gaussian splatting. Proceedings of the 2024 IEEE/CVF Conference on Computer Vision and Pattern Recognition (CVPR).

[87] Sun L.C., Bhatt N.P., Liu J.C., Fan Z., Wang Z., Humphreys T.E., Topcu U. (2024). Mm3dgs slam: Multi-modal 3D gaussian splatting for slam using vision, depth, and inertial measurements. Proceedings of the 2024 IEEE/RSJ International Conference on Intelligent Robots and Systems (IROS).

[88] Sun J., Jiao H., Li G., Zhang Z., Zhao L., Xing W. 3dgstream: On-the-fly training of 3D gaussians for efficient streaming of photo-realistic free-viewpoint videos. Proceedings of the 2024 IEEE/CVF Conference on Computer Vision and Pattern Recognition (CVPR).

[89] Liang Z., Zhang Q., Feng Y., Shan Y., Jia K. Gs-ir: 3D gaussian splatting for inverse rendering. Proceedings of the 2024 IEEE/CVF Conference on Computer Vision and Pattern Recognition (CVPR).

[90] Guédon A., Lepetit V. Sugar: Surface-aligned gaussian splatting for efficient 3D mesh reconstruction and high-quality mesh rendering. Proceedings of the 2024 IEEE/CVF Conference on Computer Vision and Pattern Recognition (CVPR).

[91] Chen Y., Lee G.H. (2024). Dogaussian: Distributed-oriented gaussian splatting for large-scale 3D reconstruction via gaussian consensus. arXiv.

[92] Zielonka W., Bagautdinov T., Saito S., Zollhöfer M., Thies J., Romero J. (2023). Drivable 3D gaussian avatars. arXiv.

[93] Duisterhof B.P., Mandi Z., Yao Y., Liu J.W., Shou M.Z., Song S., Ichnowski J. (2023). Md-splatting: Learning metric deformation from 4D gaussians in highly deformable scenes. arXiv.

[94] Wang J., Fang J., Zhang X., Xie L., Tian Q. Gaussianeditor: Editing 3D gaussians delicately with text instructions. Proceedings of the 2024 IEEE/CVF Conference on Computer Vision and Pattern Recognition (CVPR).

[95] Sturm J., Engelhard N., Endres F., Burgard W., Cremers D. (2012). A benchmark for the evaluation of RGB-D SLAM systems. Proceedings of the 2012 IEEE/RSJ International Conference on Intelligent Robots and Systems.

[96] Geiger A., Lenz P., Stiller C., Urtasun R. (2013). Vision meets robotics: The kitti dataset. Int. J. Robot. Res..

[97] Xiao J., Owens A., Torralba A. Sun3d: A database of big spaces reconstructed using sfm and object labels. Proceedings of the 2013 IEEE International Conference on Computer Vision.

[98] Chang A.X., Funkhouser T., Guibas L., Hanrahan P., Huang Q., Li Z., Savarese S., Savva M., Song S., Su H. (2015). Shapenet: An information-rich 3D model repository. arXiv.

[99] Wu Z., Song S., Khosla A., Yu F., Zhang L., Tang X., Xiao J. 3D shapenets: A deep representation for volumetric shapes. Proceedings of the 2015 IEEE Conference on Computer Vision and Pattern Recognition (CVPR).

[100] Aanæs H., Jensen R.R., Vogiatzis G., Tola E., Dahl A.B. (2016). Large-scale data for multiple-view stereopsis. Int. J. Comput. Vis..

[101] Schops T., Schonberger J.L., Galliani S., Sattler T., Schindler K., Pollefeys M., Geiger A. A multi-view stereo benchmark with high-resolution images and multi-camera videos. Proceedings of the 2017 IEEE Conference on Computer Vision and Pattern Recognition (CVPR).

[102] Knapitsch A., Park J., Zhou Q.Y., Koltun V. (2017). Tanks and temples: Benchmarking large-scale scene reconstruction. ACM Trans. Graph. (ToG).

[103] Dai A., Chang A.X., Savva M., Halber M., Funkhouser T., Nießner M. Scannet: Richly-annotated 3D reconstructions of indoor scenes. Proceedings of the 2017 IEEE Conference on Computer Vision and Pattern Recognition (CVPR).

[104] Mildenhall B., Srinivasan P.P., Ortiz-Cayon R., Kalantari N.K., Ramamoorthi R., Ng R., Kar A. (2019). Local light field fusion: Practical view synthesis with prescriptive sampling guidelines. ACM Trans. Graph. (ToG).

[105] Hedman P., Philip J., Price T., Frahm J.M., Drettakis G., Brostow G. (2018). Deep blending for free-viewpoint image-based rendering. ACM Trans. Graph. (ToG).

[106] Straub J., Whelan T., Ma L., Chen Y., Wijmans E., Green S., Engel J.J., Mur-Artal R., Ren C., Verma S. (2019). The replica dataset: A digital replica of indoor spaces. arXiv.

[107] Yao Y., Luo Z., Li S., Zhang J., Ren Y., Zhou L., Fang T., Quan L. Blendedmvs: A large-scale dataset for generalized multi-view stereo networks. Proceedings of the 2020 IEEE/CVF Conference on Computer Vision and Pattern Recognition (CVPR).

[108] Barron J.T., Mildenhall B., Verbin D., Srinivasan P.P., Hedman P. Mip-nerf 360: Unbounded anti-aliased neural radiance fields. Proceedings of the 2022 IEEE/CVF Conference on Computer Vision and Pattern Recognition (CVPR).

[109] Xiangli Y., Xu L., Pan X., Zhao N., Rao A., Theobalt C., Dai B., Lin D. (2022). Bungeenerf: Progressive neural radiance field for extreme multi-scale scene rendering. European Conference on Computer Vision.

[110] Lin L., Liu Y., Hu Y., Yan X., Xie K., Huang H. (2022). Capturing, reconstructing, and simulating: The urbanscene3d dataset. European Conference on Computer Vision.

[111] Jin H., Liu I., Xu P., Zhang X., Han S., Bi S., Zhou X., Xu Z., Su H. Tensoir: Tensorial inverse rendering. Proceedings of the 2023 IEEE/CVF Conference on Computer Vision and Pattern Recognition (CVPR).

[112] Wang Z., Bovik A.C., Sheikh H.R., Simoncelli E.P. (2004). Image quality assessment: From error visibility to structural similarity. IEEE Trans. Image Process..

[113] Huang L., Bai J., Guo J., Li Y., Guo Y. (2024). On the error analysis of 3D gaussian splatting and an optimal projection strategy. European Conference on Computer Vision.

[114] Navaneet K., Meibodi K.P., Koohpayegani S.A., Pirsiavash H. (2023). Compact3d: Compressing gaussian splat radiance field models with vector quantization. arXiv.

[115] Girish S., Gupta K., Shrivastava A. (2024). Eagles: Efficient accelerated 3D gaussians with lightweight encodings. European Conference on Computer Vision.

[116] Cheng K., Long X., Yang K., Yao Y., Yin W., Ma Y., Wang W., Chen X. Gaussianpro: 3d gaussian splatting with progressive propagation. Proceedings of the Forty-First International Conference on Machine Learning.

[117] Lee J.C., Rho D., Sun X., Ko J.H., Park E. Compact 3D gaussian representation for radiance field. Proceedings of the 2024 IEEE/CVF Conference on Computer Vision and Pattern Recognition (CVPR).

[118] Han Y., Yu T., Yu X., Xu D., Zheng B., Dai Z., Yang C., Wang Y., Dai Q. (2024). Super-NeRF: View-consistent detail generation for NeRF super-resolution. IEEE Trans. Vis. Comput. Graph..

[119] Li D., Huang S.S., Huang H. (2025). MPGS: Multi-plane Gaussian Splatting for Compact Scenes Rendering. IEEE Trans. Vis. Comput. Graph..

[120] Dai P., Xu J., Xie W., Liu X., Wang H., Xu W. (2024). High-quality surface reconstruction using gaussian surfels. ACM SIGGRAPH 2024 Conference Papers.

[121] Yu Z., Sattler T., Geiger A. (2024). Gaussian opacity fields: Efficient adaptive surface reconstruction in unbounded scenes. ACM Trans. Graph. (TOG).

